# mTORC1-selective activation of translation elongation promotes disease progression in chronic lymphocytic leukemia

**DOI:** 10.1038/s41375-023-02043-3

**Published:** 2023-09-29

**Authors:** Natasha Malik, Jodie Hay, Hassan N. B. Almuhanna, Karen M. Dunn, Jamie Lees, Jennifer Cassels, Jiatian Li, Rinako Nakagawa, Owen J. Sansom, Alison M. Michie

**Affiliations:** 1 University of Glasgow; Institute of Cancer Sciences, College of Medicine, Veterinary and Life Sciences, University of Glasgow, Glasgow, UK; 2 Immunity and Cancer Laboratory, The Francis Crick Institute, London, UK; 3 Cancer Research UK Beatson Institute; Garscube Estate, Glasgow, UK

**Keywords:** Chronic lymphocytic leukaemia, Preclinical research, Cancer models

## Abstract

Targeted deletion of *Raptor*, a component of mechanistic target of rapamycin complex 1 (mTORC1), reveals an essential role for mTORC1 in initiation/maintenance of leukemia in a CLL model, resulting from a failure for haemopoietic stem/progenitor cells (HSPCs) to commit to the B cell lineage. Induction of *Raptor*-deficiency in NSG mice transplanted with *Mx1-Raptor* CLL progenitor cells (PKCα-KR-transduced HSPCs) after disease establishment revealed a reduction in CLL-like disease load and a significant increase in survival in the mice. Interestingly in an aggressive CLL-like disease model, rapamycin treatment reduced disease burden more effectively than AZD2014 (dual mTORC1/2 inhibitor), indicating a skew towards mTORC1 sensitivity with more aggressive disease. Rapamycin, but not ibrutinib, efficiently targeted the eEF2/eEF2K translation elongation regulatory axis, downstream of mTORC1, resulting in eEF2 inactivation through induction of eEF2^T56^ phosphorylation. mTOR inhibitor treatment of primary patient CLL cells halted proliferation, at least in part through modulation of eEF2K/eEF2 phosphorylation and expression, reduced protein synthesis and inhibited expression of MCL1, Cyclin A and Cyclin D2. Our studies highlight the importance of translation elongation as a driver of disease progression and identify inactivation of eEF2 activity as a novel therapeutic target for blocking CLL progression.

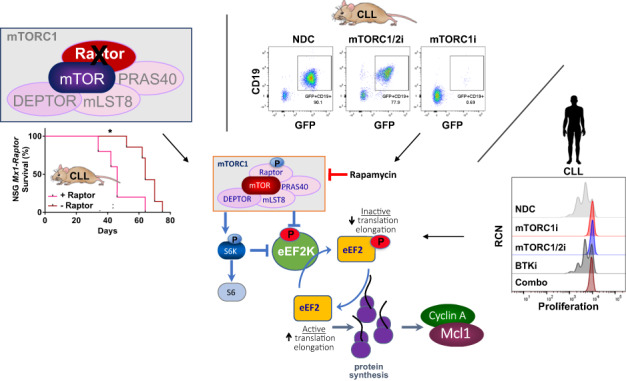

## Introduction

Clinical management of chronic lymphocytic leukemia (CLL) patients has been transformed by the introduction of targeted therapies that disrupt tumor microenvironmental signals, leading to enhanced survival rates of poor-prognostic patients, highlighting a potential curative strategy [1]. However, these treatments are not suitable for all CLL patients and the development of drug resistance has been demonstrated [2, 3]. There is an unmet clinical need for novel treatments for high-risk CLL patients. The tumor microenvironment within lymphoid organs of CLL patients promotes interaction of the leukemic clone with the stromal niche, antigen and activated-CD4^+^CD40L^+^ T lymphocytes [4–7] playing a pivotal role in enabling survival, proliferation and chemoresistance. Inhibiting the signals orchestrating these events is key to disrupting disease progression [4].

Mechanistic target of rapamycin (mTOR) plays an essential role in a multitude of cellular functions including proliferation and survival, facilitated by protein synthesis and metabolic processes [8, 9]. mTOR functions in two complexes, mTORC1 and mTORC2: RAPTOR (rapamycin TOR sensitive) is a unique and essential component of mTORC1 [8]. The critical role played by mTORC1 in positively regulating protein synthesis is highlighted by its regulation of targets p70 S6 kinase (S6K) and eukaryotic initiation factor 4E-binding proteins (4EBP1). Phosphorylation of 4EBP1 leads to the release of eIF4E enabling formation of the eIF4F complex that mediates 5’ cap-dependent translation of mRNA transcripts. S6K also contributes to translation initiation through eIF4B activation, promotion of ribosome biogenesis through activation of S6, and inhibition of eukaryotic elongation factor 2 kinase (eEF2K), a Ca^2+/^calmodulin-dependent member of the α-kinase family. eEF2K negatively regulates GTPase eukaryotic elongation factor 2 (eEF2), leading to translation elongation suppression [8, 10]. These processes are highly deregulated in transformed cells to maintain the demands of increased proliferation: the elongation stage of mRNA translation consuming almost all the energy and amino acids used during protein synthesis [10].

Conditional knockout (cKO) mouse models ablating *Raptor* expression during B cell development demonstrates the importance of mTORC1 at multiple stages of B cell lineage commitment, maturation and function [11–15]. The AKT/mTOR axis plays a key role in leukemogenesis as demonstrated in a mouse model of leukemia induced by *Pten-*loss, in which mTORC1 deletion resulted in a significant increase in survival [16]. Within CLL, the mTOR pathway is differentially modulated in CLL patients from distinct cohorts with an elevation in 4EBP1 phosphorylation in poorer prognostic CLL patients. This was coupled with an elevation in mTOR signalling in an aggressive CLL murine model [17]. Supporting these findings, mice treated with mTORC1/2-targeted inhibitors reduced CLL disease load [17, 18]. While mTORC1-selective inhibitors, or rapalogs, have shown some promise in targeting CLL through their ability to induce cell cycle arrest by modulating the mTOR/S6K pathway [19–21], a deeper understanding of the molecular processes that are deregulated in B cell malignancies downstream of mTOR is required to identify novel and potentially more selective therapeutic avenues. Here, we assessed the precise role of mTORC1 in the pathogenesis of CLL, using *Raptor-*deleted mouse models together with CLL patient samples, and identify modulation of eEF2K/eEF2 signalling and translation elongation as important regulators of CLL progression.

## Materials and methods

### Mice, primary cells and cell lines

Mice expressing the Cre/loxP system with *Raptor* (*Raptor*^*fl/fl*^) were obtained from Prof. Michael Hall (University of Basel, Switzerland) [22] and crossed with *Mx1*-cre^+/-^ or *CD19*-cre^+/-^ (Dr. Dinis Calado (Francis Crick Institute, UK)) mice (Supplementary Fig. 1). B6.SJL mice (6–10 wk old) were a source of wild type (WT) bone marrow (BM) and NOD-SCID-γc^−/−^ (NSG) mice were hosts (6–8 wk old; both sexes) for transplantation of CLL-like disease. All mice were housed at the Beatson Research Unit or Veterinary Research Facility (University of Glasgow, UK). Experimental protocols were approved by the AWERB committee and Home Office (PD6C67A47). The *Mx1* promoter is activated upon TLR3 activation [23] by inoculating *Mx1*.cre^+^-*Raptor*^*fl/fl*^ mice with 10 mg/kg TLR3 agonist poly(I:C) once every two days as indicated (GE Healthcare, WI) to induce *Raptor* cKO in the mouse (*Mx1*-*Raptor* cKO). *Mx1*.cre^-^-*Raptor*^*fl/fl*^ mice were used as controls (*Mx1.Raptor* control) [14]. *CD19* is expressed at the pro-B cell stage of B-cell development, thus targeting *Raptor-*deficiency in *CD19*-cre^+^ mice in the B-lineage only (*CD19*-*Raptor* KO) compared with *CD19*-cre^-^ mice (*CD19*-*Raptor* control) [24]. Peripheral blood was obtained, after informed consent, from patients with a confirmed diagnosis of CLL that were treatment-naïve or had received treatment but not in the preceding 3 months. The studies were approved by the West of Scotland Research Ethics Service, NHS Greater Glasgow and Clyde (UK) and carried out in accordance with the approved guidelines (REC Ref: 20/WS/0066). Linked clinical data of prognostic markers of CLL patients were recorded (Supplementary Table 1). CLL cell purity was ≥ 95% in all cases, determined by flow cytometry. Buffy coats were obtained from the Scottish National Blood Transfusion Service (UK; Ref 17 ~ 10) and mature B cells were isolated as described previously [17].

### Retroviral transduction of hematopoietic progenitors, transplantations and drug treatments

BM-derived hematopoietic progenitor cells (HPCs) isolated from *Mx1*-*Raptor* control and *Mx1*-*Raptor* cKO mice ± poly(I:C) inoculation), *CD19*-*Raptor* control and *CD19*-*Raptor* KO mice or B6.SJL WT mice, were re-suspended in 80 µl MACS buffer (2% FBS, 2 mM EDTA in PBS) and 20 µl CD117 MicroBeads (Miltenyi Biotec) to enrich LSK cells as per the manufacturers protocol. LSKs were retrovirally-transduced with either empty vector (MIEV) or kinase dead PKCα (PKCα-KR) vector which induces a CLL-like disease [25]. Cells were co-cultured with the OP9 cell line supplemented with IL-7 and Flt-3 (10 ng/ml of each; Peprotech Ltd.) until day 7–10 prior to transplantation, passaging every 2–3 days. 5 × 10^5^ cells/100 µl cells were transplanted into NSG host mice via tail vein injection to establish a CLL-like disease in vivo. Disease progression was monitored by blood sampling. Once disease was established in host mice transplanted with *Mx1-Raptor* control/cKO PKCα-KR cells, half the cohort was given 4 doses of 10 mg/kg poly(I:C) inoculations over 8 days to induce *Raptor*-excision. Secondary transplants were performed using splenic cells isolated from mice transplanted with WT PKCα-KR-transduced cells. 3 × 10^5^ splenic cells were transplanted into NSG host mice as secondary transplants. After confirmation of CLL-like disease (≥ 5% GFP^+^CD19^+^ cells in the blood), host mice were treated for up to 3 wk with AZD2014 (a gift from AstraZeneca, Cambridge, UK), rapamycin or vehicle control ensuring even disease distribution between groups. AZD2014 - 3 mg/mL in 20% Captisol (Ligand Pharmaceuticals, Inc., La Jolla, CA) was administered daily at 15 mg/kg via oral gavage; Rapamycin - Tween-80 5.2%/PEG-400 5.2% (v/v) was delivered once daily by intraperitoneal injection at 4 mg/kg [17]. Animals that failed to develop CLL-like disease were excluded from the drug treatment study.

### Statistics

Statistical analyses were carried out using GraphPad Prism 6 Software (GraphPad Software Inc., San Diego, CA). p values were determined by two-tailed students paired/unpaired *t*-test, log-rank test for Kaplan–Meier curves or mixed model ANOVA on a minimum of at least 3 biological replicates as described in individual figure legends. Biological replicates were derived from individual mice, patient samples or cell culture conditions from distinct biological samples. For drug treatment studies in vivo, power calculations were used to determine group sizes. Investigators were not blinded to sample or treatment during experiments. For experiments with primary CLL samples, no statistical methods were used to determine sample size. Mean ± SEM is shown. ^*^p$\le$0.05; ^**^p$\le$0.01; ^***^p$\le$0.001; ^****^p$\le$0.0001.

Additional methods are described in the accompanying *Supplementary Data File*.

## Results

### *Raptor* (mTORC1)-deficiency negatively impacts on normal B cell development

**Fig. 1:**
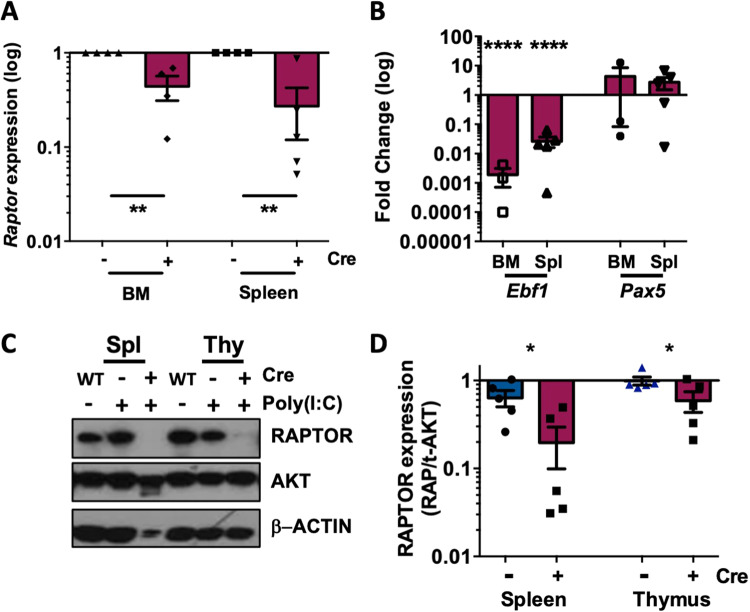
Validation of *Mx1*-*Raptor* mouse model for *Raptor*-excision. Gene expression of *Raptor* (*n* = 4) **(A)** and *Ebf1* and *Pax5* (*n* ≥ 3) **(B)** in the BM and spleen of *Mx1*-*Raptor* cKO mice (red) shown relative to *Mx1*-*Raptor* control mice, 5 weeks after treatment with poly(I:C). Gene expression is shown relative to *GusB* reference gene. **C** Representative Western blots showing protein expression levels of RAPTOR, with AKT and ß-Actin as a loading control in C57Bl/6 (WT), *Mx1*-*Raptor* control and *Mx1*-*Raptor* cKO BM, spleen, and thymus extracted from mice, after treatment with poly(I:C). **D** Quantitative protein expression of RAPTOR in the spleen and thymus of *Mx1*-*Raptor* control and *Mx1*-*Raptor* cKO mice relative to AKT (*n* ≥ 5 individual mice). All data are expressed as mean ± SEM, with *p* values were determined by two-tailed unpaired *t*-test (*p ≤ 0.05; ^**^p ≤ 0.01, ^****^p ≤ 0.0001).

*Mx1-Raptor* cKO (*Mx1*-cre^+^*Raptor*^*flfl*^) mice were inoculated with poly(I:C) and assessed 5 weeks post-treatment, comparing with inoculated *Mx1-Raptor* control (*Mx1*-cre^-^*Raptor*^*fl/fl*^) mice. A significant reduction in *Raptor* expression was noted, together with a significant decrease in *Ebf1* gene expression, but no change in *Pax5* in the BM and spleen of *Mx1-Raptor* cKO mice, suggesting an early block in B-cell development in *Mx1-Raptor* cKO mice, in agreement with previous studies (Fig. 1A, B) [12–14, 16]. Excision was also validated at the protein level with a significant reduction of RAPTOR expression in the spleen and thymus in *Mx1-Raptor* cKO mice (Fig. 1C, D). Both *Mx1-Raptor* cKO and *CD19-Raptor* KO (*CD19*-cre^+^*Raptor*^*wt/fl*^) models exhibited a significant decrease in the percentage of mature T2, marginal zone progenitor, MZ, and follicular 2 B-cells in the spleen compared to respective controls (Supplementary Figs. 2 and 3). This suggests an aberration in late B-cells upon *Raptor* (mTORC1) ablation, in agreement with previous studies [12, 13, 16].

### mTORC1 plays a fundamental role in CLL-like disease initiation in vitro

To assess the role of mTORC1 in CLL initiation, we retrovirally-transduced HPCs from *Raptor*^fl/fl^ mouse BM with kinase dead PKCα (PKCα-KR) to induce a CLL-like disease in vitro. *Mx1-Raptor* cKO HPCs transduced with MIEV (vector control) or PKCα-KR constructs did not develop B lineage cells or CLL-like disease (GFP^+^CD19^+^ population) respectively, compared to *Mx1-Raptor* control HPCs (Supplementary Fig. 4A–C). Myeloid lineage commitment was spared: *Raptor-*deficiency led to a significant increase in the percentage of CD11b^+^ myeloid cells in MIEV and PKCα-KR transduced co-cultures with time, as we and others have shown previously in vivo (Supplementary Fig. 4A, D, E) [12–14]. In contrast, PKCα-KR-retrovirally-transduced *CD19-Raptor* KO HPCs generated CLL-like disease (GFP^+^CD19^+^ population) similar to *CD19-Raptor* control HPCs in vitro (Supplementary Fig. 4F, G). These findings underline the essential role played by mTORC1-signalling during B-lineage commitment and demonstrate an HPC-mediated block in CLL-like disease initiation with *Raptor*-deficiency.

### mTORC1 is important for CLL-like disease proliferation in vitro and in vivo

**Fig. 2:**
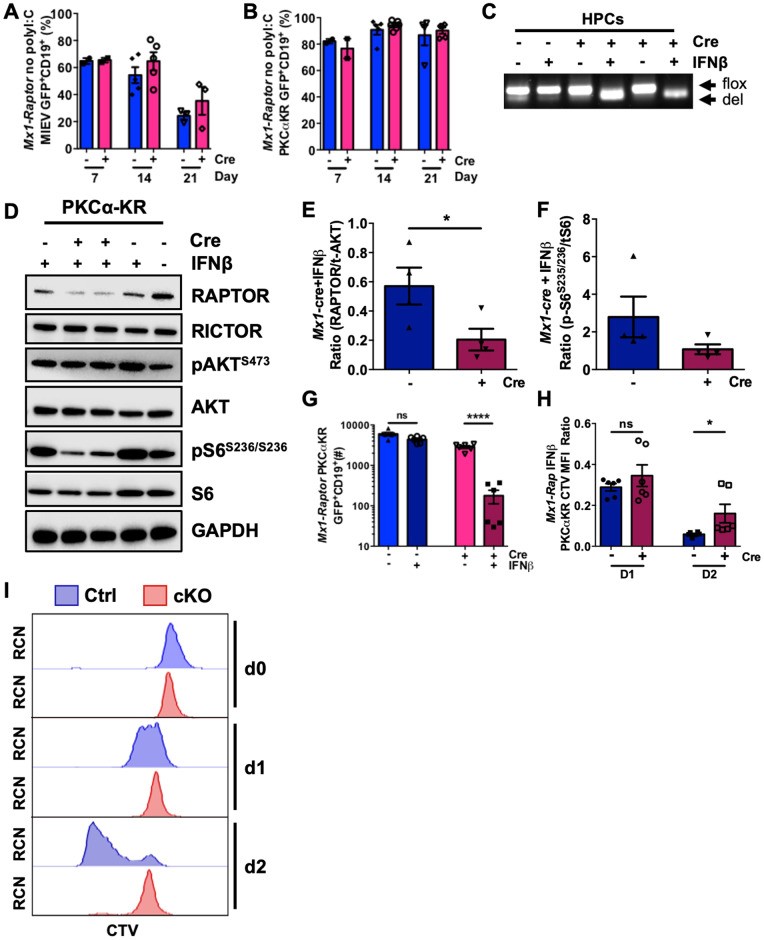
Inducible model of *Raptor*-deficiency abrogates CLL-like disease maintenance in vitro. Bar graphs showing the percentage of CD19^+^GFP^+^ *Mx1-Raptor* control or *Mx1-Raptor* cKO MIEV **(A)** and *Mx1-Raptor* control or *Mx1-Raptor* cKO PKCα-KR **(B)** cells at D7 (*n* = 2), D14 (*n* = 4), and D21 (*n* = 3/4) with no induction of *Raptor* excision. Data expressed as mean±range/SEM of independent cell cultures from individual mice. **C** DNA agarose gel showing excision of *Raptor* in *Mx1-Raptor* cKO (no polyI:C) HPCs treated with 200U IFNβ for 24 h (deletion of exon 6 (del)), compared with *Mx1-Raptor* control with or without 200U IFNβ for 24 h (flox), and untreated *Mx1-Raptor* cKO (no polyI:C) HPCs. Cells were assessed 72 h post-treatment. Representative Western blot **(D)** and bar graphs showing expression of RAPTOR/t-AKT **(E)**, pS6^S235/236^/tS6 **(F)** of *Mx1-Raptor* control or cKO PKCα-KR (*n* = 4) cells treated with IFNβ and assessed 72 h post treatment. **G** Cell number (*n* = 6) of *Mx1-Raptor* control or cKO PKCα-KR cells either untreated (light blue and pink bars) or treated (blue and red bars) with 200U IFNβ for 24 h and assessed at 72 h post treatment. Bar graphs **(H)** and representative plots **(I)** showing CTV MFI at d0, d1 and d2 of *Mx1-Raptor* control or cKO PKCα-KR cells (*n* = 6) treated with 200U IFNβ for 24 h and assessed at 72 h post treatment. Relative cell number (RCN). All data are expressed as mean ± SEM, with *p* values were determined by two-tailed unpaired *t*-test (*p ≤ 0.05, ^****^p ≤ 0.00001).

*CD19-Raptor* KO cells displayed a significant reduction in proliferation when transduced with PKCα-KR compared to *CD19-Raptor* control PKCα-KR cells after 24 h in vitro (Supplementary Fig. 5A, B). Moreover, there was a significant reduction in migration towards SDF-1 in *CD19-Raptor* KO PKCα-KR cells compared to *CD19-Raptor* control PKCα-KR cells (Supplementary Fig. 5C, D). To investigate these data further, we assessed the role of *Raptor* in CLL-like disease proliferation and maintenance in vitro using the inducible cKO model (*Mx1-Raptor*). In the absence of *Raptor* excision, both *Mx1-Raptor* control and *Mx1-Raptor* cKO HPCs developed into B-cells or a CLL-like disease (Fig. 2A, B). To assess disease maintenance in the absence of *Raptor* (mTORC1) in vitro, CLL-like co-cultures were treated with interferon β (IFNβ) to activate the TLR3 receptor. Confirmation of *Raptor* excision after IFNβ treatment, was indicated by reduced levels of pS6^S235/236^ in *Mx1-Raptor* cKO PKCα-KR cells compared to *Mx1-Raptor* control PKCα-KR cells (Fig. 2C–F). Furthermore, co-culture of PKCα-KR cells revealed a significant decrease in the GFP^+^CD19^+^ cell number and reduced proliferation of *Mx1-Raptor* cKO CLL-like cells (and not *Mx1-Raptor* control cells) upon IFNβ treatment (Fig. 2G–I), supporting the role of mTORC1 in promoting leukemia propagation [13].

**Fig. 3:**
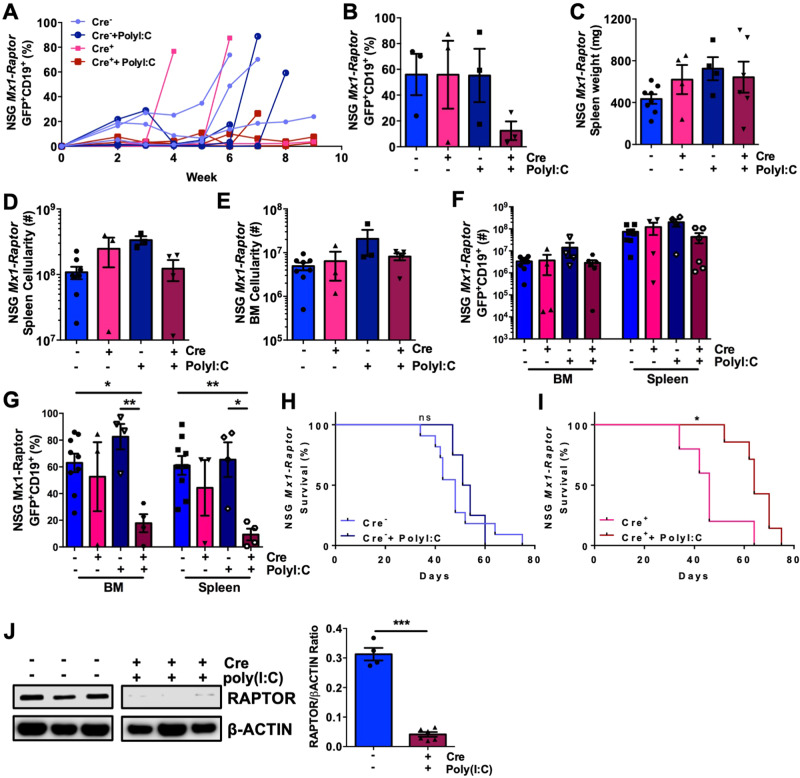
NSG mice with established CLL-like disease exhibit a decrease in disease load with induced *Raptor*-deficiency in vivo. NSG mice were transplanted with *Mx1-Raptor-*PKCα-KR cells to establish CLL-like disease. Graphs show the percentage of GFP^+^CD19^+^ CLL-like disease in transplanted NSG blood samples taken weekly (representative *n* = 3 individual mice shown/arm of n ≥ 6/arm) **(A)** and at end point **(B)** from *Mx1-Raptor* control PKCα-KR cells (pale blue), *Mx1-Raptor* control PKCα-KR cells+polyI:C after disease development (dark blue), *Mx1-Raptor* cKO PKCα-KR cells (pink) or *Mx1-Raptor* cKO PKCα-KR cells+polyI:C after disease development (red). Spleen weight (mg) **(C)**, spleen **(D)** and BM **(E)** cellularity of NSG mice transplanted with either *Mx1-Raptor* control PKCα-KR cells (light blue, *n* = 8), *Mx1-Raptor* cKO PKCα-KR cells (pink, *n* = 3), *Mx1-Raptor* control PKCα-KR cells+polyI:C after disease development (dark blue, *n* = 4), or *Mx1-Raptor cKO* PKCα-KR cells+polyI:C after disease development (red, *n* = 5). Cell number **(F)** and percentage **(G)** of GFP^+^CD19^+^ cells in BM and spleen of NSG mice transplanted with either *Mx1-Raptor* control PKCα-KR cells (*n* = 8), *Mx1-Raptor* cKO PKCα-KR cells (*n* = 3), *Mx1-Raptor* control PKCα-KR cells+polyI:C after disease development (*n* = 4) or *Mx1-Raptor* cKO PKCα-KR cells+polyI:C after disease development (*n* = 5). All data are expressed as mean ± SEM, with *p* values were determined by an ordinary one-way ANOVA with Tukey’s multiple comparisons test (*p ≤0.05, **p ≤ 0.001). Survival graphs comparing the percentage survival between NSG mice transplanted with *Mx1-Raptor* control PKCα-KR cells with (*n* = 4) or without (*n* = 11) polyI:C inoculation (Control; **H**), and *Mx1-Raptor* cKO PKCα-KR cells with (*n* = 5) or without (*n* = 7) polyI:C inoculation (cKO; **I**) after disease development. *p* values were determined by log rank test for Kaplan–Meier curves (*p ≤ 0.05)**. J** NSG mice were transplanted with *Mx1-Raptor-*PKCα-KR cells to establish CLL-like disease. Representative Western blot of splenic cells (left) and densitometry of RAPTOR expression relative to b-Actin loading control (right) in *Mx1-Raptor* control PKCα-KR cells (light blue, *n* = 4), or *Mx1-Raptor* cKO PKCα-KR cells+poly(I:C) after disease development (red, *n* = 6). These blots are the same as those shown in Supplementary Fig. 14A, showing the same β-Actin loading control. *p* values were determined by two-tailed unpaired *t*-test for bar graphs (^***^p ≤ 0.0001). All data are expressed as mean ± SEM.

To assess whether mTORC1 plays a similar role in disease maintenance in vivo, NSG mice were transplanted with PKCα-KR retrovirally-transduced *Mx1-Raptor* control or *Mx1-Raptor* cKO HPCs. After disease establishment, control and cKO mice were treated with poly(I:C) to assess disease maintenance with *Raptor-*deficiency. Blood sampling demonstrated a reduced percentage of GFP^+^CD19^+^ cells in mice transplanted with *Mx1-Raptor* cKO PKCα-KR cells and treated with poly(I:C), while disease load continued to increase in mice transplanted with *Mx1-Raptor* control PKCα-KR cells±poly(I:C) treatment and *Mx1-Raptor* cKO PKCα-KR cells in the absence of poly(I:C) (Fig. 3A, B and Supplementary Fig. 6). While no change was noted in splenic weight or total spleen and BM cellularity (Fig. 3C–E), a decrease in disease load was seen in the spleens of mice transplanted with *Mx1-Raptor* cKO PKCα-KR cells with poly(I:C) treatment compared to *Mx1-Raptor* cKO mice without poly(I:C) (Fig. 3F). In addition, there was a significant decrease in the percentage of GFP^+^CD19^+^ cells in the BM and spleen of poly(I:C) treated mice transplanted with *Mx1-Raptor* cKO PKCα-KR cells compared with *Mx1-Raptor* control PKCα-KR cells (Fig. 3G). A significant increase was seen in survival of poly(I:C) treated mice transplanted with *Mx1-Raptor* cKO PKCα-KR cells compared to untreated mice transplanted with *Mx1-Raptor* cKO PKCα-KR cells (Fig. 3H, I). Validating *Raptor-*deficiency, spleens showed a significant reduction in Raptor expression, with barely detectable expression of Raptor in the poly(I:C) treated mice transplanted with *Mx1-Raptor* cKO PKCα-KR cells (Fig. 3J). These studies demonstrate that leukaemic cells require mTORC1 signalling for tumour expansion, and that mTORC1 plays a vital role in driving leukemia progression in our CLL mouse model in vivo.

**Fig. 4:**
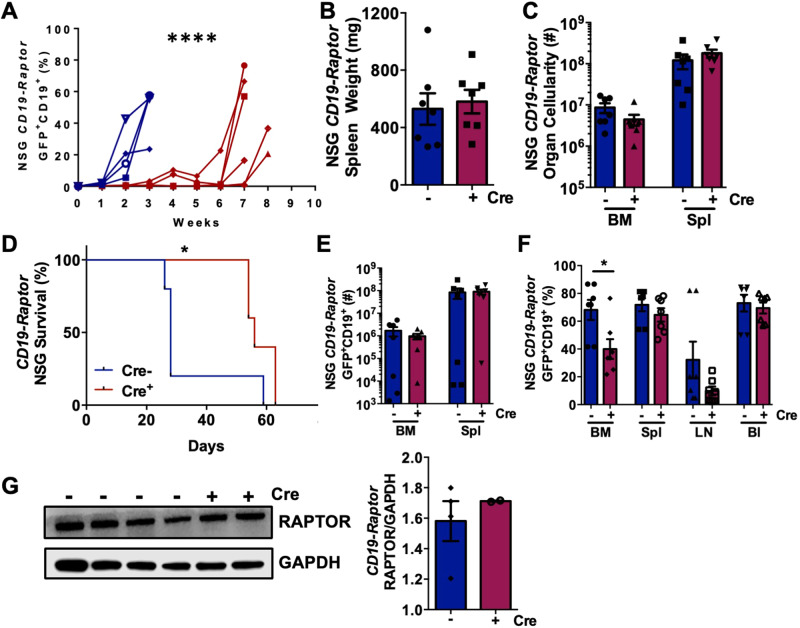
*Raptor*-deficiency in CD19^+^ CLL-like cells increases survival in CLL-like disease in vivo. **A** Weekly blood samples were taken and assessed for percentage of GFP^+^CD19^+^ CLL-like disease in NSG mice transplanted with *CD19-Raptor* control (blue) or cKO (red) PKCα-KR cells (*n* = 6 individual mice/arm). *p* values were determined by a two-way ANOVA with Sidak multiple comparisons test (^****^p ≤ 0.00001). Spleen weight (mg) **(B)** and BM and spleen **(C)** cellularity of NSG mice transplanted *CD19-Raptor* control or cKO PKCα-KR cells (*n* = 7/arm). **D** Survival graph comparing the survival of NSG mice transplanted with *CD19-Raptor* control (blue) or cKO (red) PKCα-KR cells (*n* = 5/arm). Cellularity of GFP^+^CD19^+^ cells in the BM and spleen **(E)** and percentage of GFP^+^CD19^+^ cells in BM, spleen, LN and blood **(F)** of NSG mice transplanted with *CD19-Raptor* control (blue) or cKO (red) PKCα-KR cells (*n* = 7/arm). Representative Western blot **(G)** and protein expression ratio of Raptor and GAPDH (loading control) in the spleen of NSG mice that were transplanted with *CD19-Raptor* control (*n* = 4) or cKO PKCα-KR cells (*n* = 2) for up to 7-8 weeks. Data are expressed as mean ± SEM. *p* values were determined by log rank test for Kaplan–Meier survival curves or two-tailed unpaired *t*-test for bar graphs (^*^p ≤ 0.05, ^****^p ≤ 0.00001).

Mice transplanted with *CD19-Raptor* KO cells exhibited a 4-5 week delay in GFP^+^CD19^+^ disease burden in the blood compared with *CD19-Raptor* control mice (Fig. 4A), supporting the above findings that *Raptor* has a non-redundant role in disease progression (Fig. 3). Although spleen weight, BM and spleen organ cellularity were unchanged, a significant increase was seen in survival of mice transplanted with *CD19-Raptor* KO PKCα-KR cells compared to *CD19-Raptor* control cells (Fig. 4B–D). While a significant decrease in percentage of GFP^+^CD19^+^ CLL-like cells was noted in the BM, with a similar trend in the lymph node (LN) of mice transplanted with *CD19-Raptor* KO PKCα-KR cells compared to controls, disease eventually accumulated, resulting in similar disease load in the spleen and blood as observed in the controls (Fig. 4E, F and Supplementary Fig. 7). This was due to the emergence of cells that escaped *Raptor*-excision, indicated by expression of RAPTOR in the spleens of mice transplanted with *CD19-Raptor* KO PKCα-KR cells (Fig. 4G). Collectively these data indicate that mTOR inhibition may provide a valid therapeutic option to target disease progression.

### Secondary transplantation enhanced rapamycin sensitivity of leukemia in vivo

**Fig. 5:**
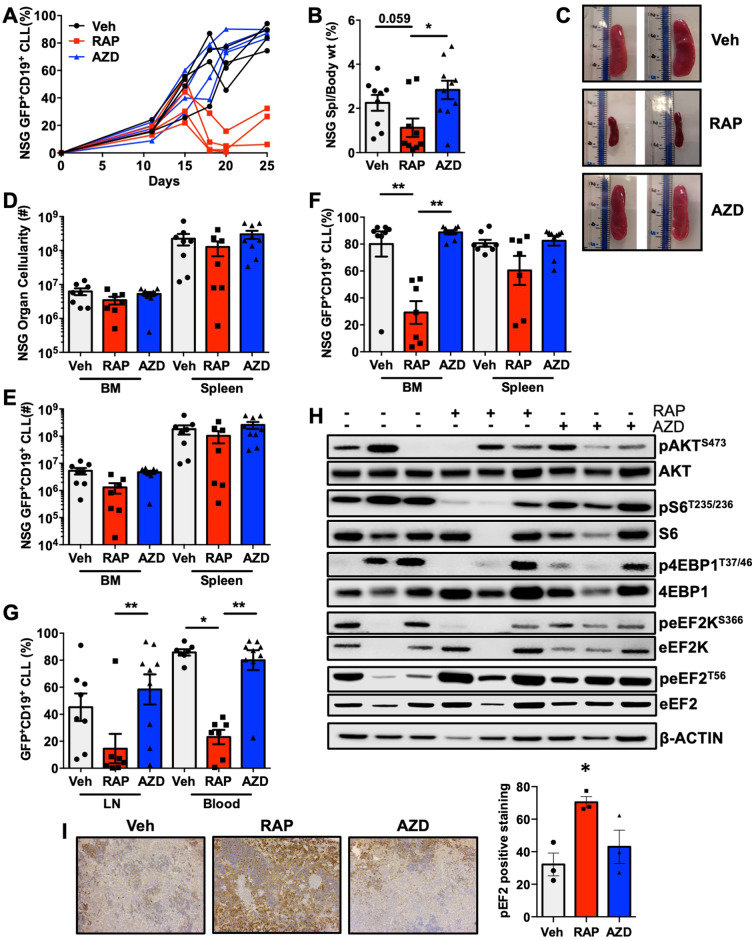
Rapamycin decreases CLL-disease load in an aggressive CLL-like model in vivo. Secondary transplants were generated in host mice by transplantation of PKCα-KR cells from spleens of NSG mice with ≥95% CLL-like disease. **A** Weekly blood samples (representative, *n* = 4/arm) were taken and assessed for percentage of GFP^+^CD19^+^ CLL-like disease in mice with established disease and treated with either Veh, RAP or AZD2014 as indicated. **B** Percentage of spleen/body weight of drug-treated NSG mice. **C** Spleens from mice with established disease, treated with vehicle control (Veh; captisol, grey), rapamycin (RAP, red) or AZD2014 (AZD, blue) (*n* ≥ 9/arm). **D** Total cellularity of the BM and spleen in drug treated NSG mice with a CLL-like disease. Cellularity **(E)** and percentage **(F)** of GFP^+^CD19^+^ CLL-like cells in the BM and spleen together with the percentage **(G)** of GFP^+^ CD19^+^ CLL-like cells in the LN and blood of drug treated NSG mice with disease (*n* ≥ 7/arm). **H** Representative Western blot of proteins as indicated, using samples of spleens from drug treated NSG mice with disease (*n* = 3/arm). **I** Splenic tissue from treated mice were stained for peEF2^T56^ by IHC and the staining was quantified (*n* = 3/arm). All data are expressed as mean ± SEM, with *p* values were determined by an ordinary one-way ANOVA with Tukey’s multiple comparisons test (*p ≤0.05, **p ≤ 0.001).

To assess mTOR sensitivity of aggressive CLL-like disease, we performed secondary transplants, using splenic cells from mice carrying CLL-like disease (≥ 95%). Upon disease establishment, mice were treated with vehicle control, rapamycin (allosteric mTORC1 inhibitor; 4 mg/kg), or AZD2014 (Vistusertib; competitive dual mTORC1/2 inhibitor; 15 mg/kg) daily for 3 wk [17, 26]. Blood samples from mice treated with rapamycin exhibited a clear reduction in GFP^+^CD19^+^ CLL-like cells compared with AZD2014 and vehicle treated mice (Fig. 5A & Supplementary Fig. 8). A reduced spleen size was seen in mice treated with rapamycin, however there were no changes in mononuclear cell BM and splenic cellularity (Fig. 5B–D). A significant reduction in the percentage of GFP^+^CD19^+^ cells was seen in rapamycin-treated mice in the BM and blood, with a similar trend in the spleen and LN, and a significant decrease in the percentage of disease in the BM, LN and blood of mice with rapamycin treatment (Fig. 5E–G). These findings suggest that CLL-like disease is more sensitive to rapamycin than AZD2014 in secondary transplanted mice.

Supporting our previous in vitro findings [17], decreases were observed in the pAKT^S473^, p4EBP1^T37/46^ and pS6^S235/S236^ signals in splenic tissue isolated from mice treated with drug for 3 weeks, with trends seen in pAKT^S473^ in AZD2014 treated mice, and in p4EBP1^T37/46^ and pS6^S235/S236^ signals in both rapamycin and AZD2014 treated mice compared with controls as expected, however these did not reach significance due to in vivo variability (Fig. 5H and Supplementary Fig. 9A–C). Analysing additional downstream mTORC1 targets to assess the mechanism driving rapamycin sensitivity, a significant decrease in peEF2K^S366^ was noted both with rapamycin and AZD2014 treatment, accompanied by an increase in peEF2^T56^ upon treatment with rapamycin, which when analysed by IHC in splenic tissue was significant in the rapamycin-treated arm, compared to vehicle control (Fig. 5H, I and Supplementary Fig. 9D, E). These data suggest a possible role for eEF2K/eEF2 signalling in driving an aggressive leukemia model and demonstrate that targeting mTORC1-eEF2K signalling can inactivate eEF2 and potentially deregulate protein translation elongation.

### mTORC1-eEF2K/eEF2 signalling is vital for proliferation in primary CLL cells

**Fig. 6:**
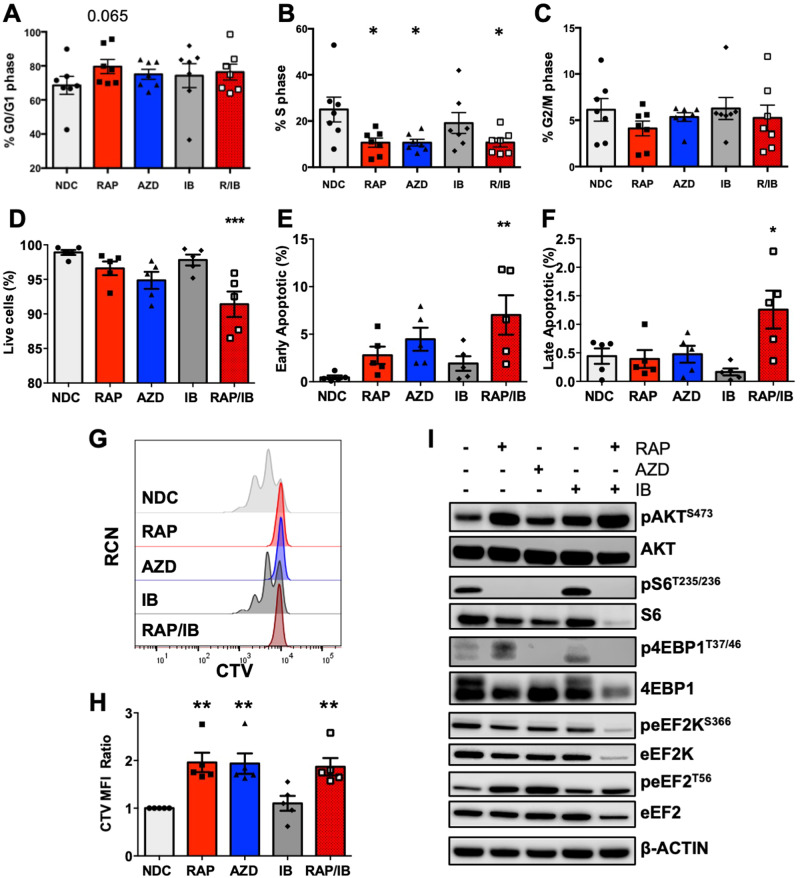
mTOR inhibitors attenuate proliferation of primary CLL patient cells and modulate eEF2K/eEF2 signalling. CLL cells were co-cultured for 5–8 days with CD40L-expressing NTL cells with 15 ng/ml IL21 to induce proliferation. Data show averages of 5 individual CLL patients. Graphs show G0/G1 phase **(A)**, S phase **(B)**, and G2/M phase **(C)** of cell cycling, excluding sub G0/G1 cells, in CLL patient samples treated with either no drug control (NDC, light grey), rapamycin (RAP, red), AZD8055 (AZD, blue), ibrutinib (IB, dark grey), or RAP/IB combination (dark red). The gating strategy for this analysis is shown in Supplementary Fig. 11. Graphs showing an average of the percentage of live cells **(D)**, early apoptotic **(E)** and late apoptotic **(F)** treated primary CLL cells as indicated. Representative half offset histogram plot showing the CTV mean fluorescence intensity (MFI; **G)**, and graph showing the CTV MFI ratio **(H)** of CLL patient samples treated as indicated (*n* = 5/arm). **I** Representative Western blot shown of protein phosphorylation/expression as indicated in primary CLL cells treated with rapamycin (RAP), AZD8055 (AZD), ibrutinib (IB), RAP/IB combination or NDC. Blots shown are representative of 5 individual CLL patient samples. All data are expressed as mean ± SEM, with *p* values were determined by an ordinary one-way ANOVA with Dunnett multiple comparisons test (*p ≤ 0.05, ^**^p ≤ 0.001, ***p ≤ 0.0001).

Human CLL models were treated with rapamycin and AZD8055 (a pharmacophore of AZD2014 (ref. [26])). AZD2014 was used in the animal studies because it has a longer half-life in vivo [17, 26]. In proliferating primary CLL cells (NTL-CD40L/IL21 co-culture), mTOR-selective inhibitors induced a significant decrease in the S phase of cell cycle alone and in the RAP/IB combination compared to ibrutinib (IB) or NDC controls, indicative of an mTORC1-mediated reduction in cell cycle (Fig. 6A–C, Supplementary Fig. 10). Supporting these data, mTOR inhibitors induced a significant decrease in the S and G_2_/M phases of the cell cycle in MEC1 cells (Supplementary Fig. 11A–D). IB induced a significant decrease in the G_2_/M phase in MEC1 cells, highlighting the MEC1 sensitivity to BTK-mediated signals compared with CD40L/IL21-induced proliferating CLL cells (Supplementary Fig. 11D). Although a significant reduction in viability was noted in MEC1 cells treated with rapamycin and AZD8055 alone, mirrored by an increase in early and late apoptosis with AZD8055 treatment (Supplementary Fig. 11E–H), a significant increase in apoptosis was seen in the combination arm (RAP/IB) only in primary CLL patient samples, supporting previous studies that rapamycin alone does not induce primary CLL cell death (Fig. 6D–F) [17, 20]. Finally, a significant decrease in MEC1 and primary CLL proliferation was noted upon rapamycin, AZD8055, and RAP/IB combination treatment compared to NDC (Fig. 6G, H & Supplementary Fig. 11I, J). IB alone did not inhibit primary CLL cell proliferation, however it did reduce MEC1 proliferation, again highlighting the differential drug responses between MEC1 and primary CLL cells. These studies underscore the importance of combining mTORC1 inhibition with additional therapies such as ibrutinib to induce CLL apoptosis.

AZD8055 treatment resulted in significant decreases in pAKT^S473^ and p4EBP1^T37/46^ signals in primary CLL cells, while there was a significant decrease in pS6^S332/336^ with rapamycin and AZD8055 in CLL patient samples (Fig. 6I & Supplementary Fig. 12A–C). Of note, RAP treatment resulted in a significant upregulation of pAKT^S473^, indicative of the mTORC1-mediated feedback inhibition being released upon mTORC1 inhibition (Fig. 6I & Supplementary Fig. 12A) [17, 27]. IB did not alter mTOR-mediated signals in CLL cells co-cultured with CD40L/IL21 co-culture. Mirroring the phosphorylation events seen in splenocytes of PKCα-KR-transduced *Mx1-Raptor* cKO transplanted mice (Fig. 5H), there was a significant decrease in peEF2K^S366^ in samples treated with RAP or AZD8055, which was enhanced in the RAP/IB combination, together with an increase in peEF2^T56^ in CLL patient samples when treated with RAP or AZD8055, which reached significance with AZD8055 treatment (Fig. 6I & Supplementary Fig. 12D, E). In mice transplanted with *Mx1-Raptor-*PKCα-KR cells and then treated with polyI:C after establishment of CLL-like disease, an increase in peEF2^T56^ was observed in the absence of *Raptor*, and a significant decrease in Cyclin A expression, a protein associated with transition through the S-G_2_/M phase of the cell cycle (Supplementary Fig. 13A). An upregulation of peEF2^T56^ was also seen in response to RAP treatment in PKCα-KR co-cultures in vitro, mirroring the signalling events observed in vivo (Fig. 5H), together with a significant downregulation of Cyclin A and Mcl1 expression, the latter associated being a marker of poor prognosis in CLL (Supplementary Fig. 13B) [28]. Furthermore RAP alone, and AZ/IB combination reduced eEF2 protein expression in PKCα-KR co-cultures in vitro (Supplementary Fig. 13B). These results suggest that pharmacological targeting of eEF2K/eEF2 may disrupt protein synthesis in CLL cells.

### Inhibition of mTORC1-eEF2 signalling reduces protein synthesis in human CLL cells

**Fig. 7:**
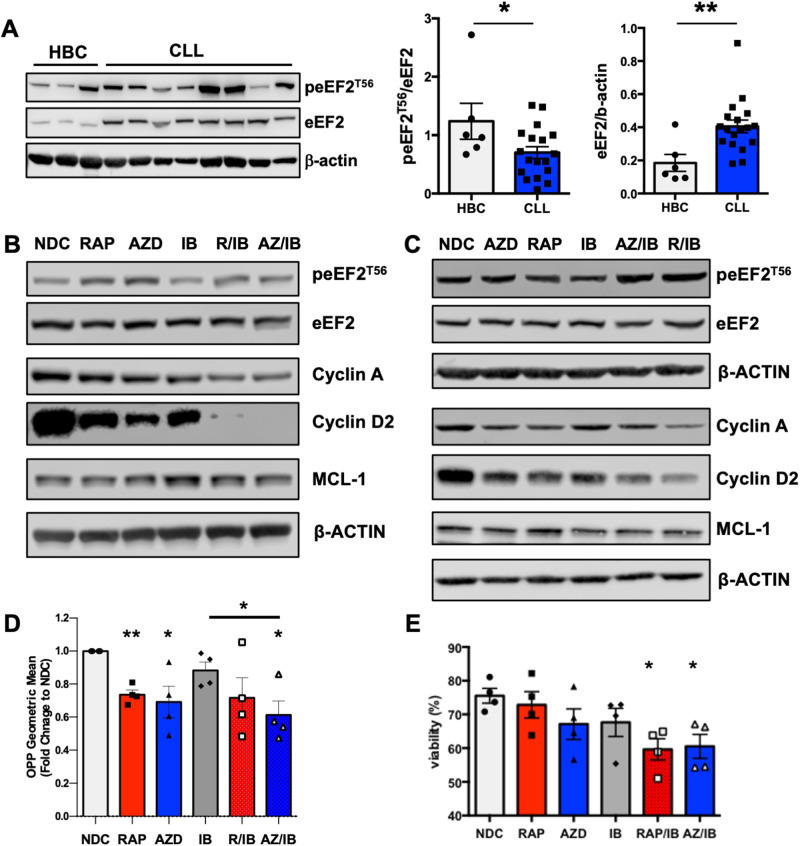
eEF2^T56^ phosphorylation/expression is modulated and protein synthesis reduced, by mTORC1 inhibition of translation elongation in primary CLL cells. **A** Protein lysates were prepared from primary CLL cells freshly isolated from the peripheral blood of consented patients (*n* = 18) and healthy B cells (HBC; *n* = 6) isolated from buffy coats. Western blots were performed and probed as indicated. Data are expressed as mean ± SEM, with *p* values were determined by two-tailed unpaired *t*-test (*p ≤ 0.05; ^**^p ≤ 0.01). **B** MEC1 cells were treated with drugs for 24 h or (**C**) proliferating CLL cells are co-cultured for 5 days with NTL-CD40L/IL21 in the presence of drugs as indicated. Representative Western blots are shown. Blots shown are representative of *n* ≥ 3 individual biological replicates. **D** OPP assays were performed on drug-treated proliferating CLL cells to assess protein synthesis (*n* = 4). Data are expressed as mean ± SEM. p values were determined by one-way ANOVA, not corrected for multiple comparisons (*p ≤ 0.05, **p ≤ 0.001). **E** Graphs showing an average of the percentage of viable cells in treated primary CLL cells (*n* = 4). Data are expressed as mean ± SEM. *p* values were determined by an ordinary one-way ANOVA with Dunnett multiple comparisons test (*p ≤ 0.05).

Analysis of primary CLL cells isolated from peripheral blood fresh ex vivo, revealed that eEF2 expression is significantly upregulated, while peEF2^T56^ is significantly downregulated in CLL cells compared with healthy B cells (Fig. 7A). MEC1 or proliferating primary CLL cells treated with RAP or AZD8055 alone or in combination with IB, showed elevated peEF2^T56^ with mTOR-selective inhibitors, indicating an inactivation of the eEF2K/eEF2 axis and a reduction in translation elongation, which did not occur with IB alone (Fig. 7B&C & Supplementary Fig. 14). Furthermore, the combination of AZ/IB resulted in a significant reduction of eEF2 expression in MEC1 and proliferating primary CLL cells (Supplementary Fig. 15A&B), mirroring the finding in PKCα-KR co-cultures. In addition, the expression of Cyclin A, Cyclin D2 and MCL1 was reduced in human CLL models when treated with mTOR inhibitors, reaching significance when combined with IB (Fig. 7B, C & Supplementary Fig. 14). Supporting this, a significant inhibition of protein synthesis (OPP assay) was noted in primary CLL cells with mTOR inhibitor-containing treatments, while IB alone had no effect (Fig. 7D), and both combination treatments (RAP/IB and AZ/IB) significantly reduced primary CLL cell viability (Fig. 7E). Collectively these data demonstrate the importance of mTORC1 in the regulation of protein synthesis at least in part through translation elongation, independently of IB -sensitive pathways, and identify the eEF2K/eEF2 signalling axis as a promising therapeutic target in CLL.

## Discussion

We highlight for the first time, the importance of the eEF2K/EF2 axis in regulating protein translation elongation downstream of mTORC1-regulated pathways in CLL models which, through an elevation eEF2 expression and reduced negative regulation of eEF2, enables the generation of proteins involved in cell survival and proliferation. Furthermore, we have identified enhanced sensitivity of aggressive leukemic cells to rapamycin, compared with the dual mTOR kinase inhibitor AZD2014. Translation elongation is an immensely understudied area of research particularly in B-cell malignancies/CLL, despite it representing a fertile space to identify novel targets for therapeutic intervention in a key process driving oncogenesis. This in turn will promote the development of compounds that lack the broad-ranging side effects that occur with rapalogs.

*Raptor*-deficient models revealed that PKCα-KR-transduction was unable to rescue the B-lineage commitment block caused by *Raptor-*deficiency and failed to initiate CLL development in *Mx1-Raptor* cKO-PKCα-KR cells. mTORC1 also plays an essential role in the leukaemia propagation in T-ALL and AML [13, 29]. Furthermore, disease maintenance was negatively impacted upon *Raptor* deletion in vivo, resulting in an increase in survival in our CLL mouse models. Kalaitzidis et al., demonstrated that *PTEN*-loss leads to MPN due to mTORC1 activation, which was reversed by inducing *Raptor* loss after MPN, suggesting a fundamental role of mTORC1 in leukemia progression [16]. Collectively, these findings support the use of mTOR inhibitors as a therapeutic option in leukemia. While *CD19-Raptor* KO PKCα-KR transplanted mice initially displayed increased survival this was not sustained, with a delayed increase in disease load in the blood of mice transplanted with *CD19-Raptor* KO PKCα-KR cells. Further assessment revealed a re-emergence of Raptor expression due to an inefficient knockout in the *CD19*-cre-loxP model, leading to ‘escaped deletions’ and subsequent disease re-population: the *CD19*-cre model induces excision with 80–93% efficiency [30]. Inefficient excision of *Raptor* may also contribute to the low level of disease observed in the organs of some *Mx1-Raptor* cKO PKCα-KR transplanted mice treated with poly(I:C), however as disease onset did not routinely occur, this indicates that the excision efficiency in this model was higher.

We have previously shown that targeting transplanted PKCα-KR CLL-like disease with AZD8055 or AZD2014 more robustly reduces disease compared with rapamycin in primary PKCα-KR transplanted mice [17]. Using an aggressive secondary transplant leukemia model revealed that AZD2014 was inferior compared to RAP in decreasing disease load in vivo. The rapalog everolimus has previously been demonstrated to elicit a modest clinical benefit in a Phase II clinical trials in CLL patients [21]. A Phase II clinical trial is currently recruiting relapsed/refractory CLL and Richter transformation patients to test a novel triple therapy (DTRM-555), combining a novel BTK inhibitor (DTRMWXHS-12), everolimus and immunomodulator pomalidomide (NCT04305444). Our results support the inclusion of everolimus with a BTK inhibitor in the treatment of aggressive CLL cohorts, as we show an inhibition of eEF2K/EF2 signalling axis in an aggressive B cell leukemia model in vivo in the presence of rapamycin, which is enhanced in combination with  IB, leading to reduction in cell survival.

A phase II clinical trial in refractory renal cancer demonstrated that AZD2014 treated patients exhibited a shorter progression-free survival and increased disease progression compared to patients treated with everolimus [31], akin to our findings. In an APC-deficient mouse model of colorectal cancer (CRC), rapamycin treatment reduced tumor growth and increased survival of mice [32]. Of note, analysis of tissues CRC patient tumor samples revealed that low eEF2K activity has been associated with a worse prognosis of patients [33, 34], indicating that stratifying patients by developing robust pharmacological biomarkers relating to translation elongation may enable more personalised treatment options for cancer patients. This is particularly important in CLL patients that exhibit substantial heterogeneity in their response to treatments.

Elevated protein synthesis is essential for driving oncogenesis, enabling increased survival and proliferation through the generation of proteins such as MCL1, MYC and cell cycle regulators. mTORC1 plays a central role in regulating protein synthesis, with suppression of translation limiting tumorigenesis [10, 32, 33, 35]. We showed that poor prognostic CLL cells have upregulated p4EBP1, indicative of elevated cap-dependent translation [17]. Supporting studies demonstrated that BCR crosslinking on CLL cells resulted in elevated expression of eIF4A and eIF4GI (part of the eIF4F complex) and drives MYC and MCL1 translation [36, 37]. Furthermore, the phytochemical phenethylisothiocyanate can elicit global inhibition of mRNA translation, including *MYC*, in CLL cells in part through elevation in eIF2α phosphorylation, suggesting that protein translation can be therapeutically targeted [38]. Here, we show that eEF2K/eEF2 activity and expression are modulated in primary proliferating CLL cells, aligning with the data from the mouse model, and propose that translation elongation is a novel therapeutic target in CLL, through inhibition of eEF2 activity [33, 39–41]. Of note, changes in eEF2 expression/phosphorylation in primary CLL cells isolated fresh ex vivo indicate that peripheral CLL cells possess elevated eEF2 activity and are primed to proliferate in comparison with normal healthy B cells, with treatments containing mTOR inhibitors reducing eEF2 expression in CLL cells. Interestingly, combination of IB with AZD8055 further reduced the expression of eEF2 in our CLL models. It is well established that the eEF2K/eEF2 axis is regulated by numerous signaling pathways [42], so developing rational drug combinations to target these proteins may form therapeutic options in the future. Translation elongation is activated under conditions of stress including nutrient/energy depletion, which can lead to a cytoprotective effect in tumour cells [42]. The potential to therapeutically target translation elongation is exemplified by a study using nelfinavir, an aspartyl protease inhibitor, a treatment of HIV patients, which selectively activates eEF2K independently of mTORC1 suppressing translation elongation [43].

PKCα-KR CLL-like cells were responsive to IB treatment, as indicated by decreased Cyclin A expression, while proliferating human CLL cells did not illicit protein expression/synthesis responses to IB. Primary CLL cell proliferation was driven by CD40L/IL21 stimulation in this study, which occurs independently of BTK, while the PKCα-KR CLL mouse model exhibits activated BCR signalling with sensitivity to IB [17, 44]. In addition, while PKCα-KR CLL exhibits increased RAP sensitivity, in terms of reducing tumour load in vivo, all CLL models displayed similar functional responses to AZD8055 and RAP. In line with this, the mouse model showed elevated peEF2^T56^ in response to RAP, AZD8055, or RAP/IB, although this was not significant. Of note, the RAP/IB combination appeared to be more effective at inhibiting eEF2 activity, indicated by an elevation in peEF2^T56^, with RAP-containing treatments consistently inhibiting MCL1 and Cyclin A expression, supporting the importance of targeting of signals downstream of mTORC1. As IB does not influence eEF2K/eEF2 signalling or protein synthesis in primary CLL cells, our results suggest that a combination of IB with nelfinavir to target both BCR-mediated signalling and translation elongation may provide enhanced clinical benefit to CLL patients.

Collectively, we have identified a novel mTORC1-biased mechanism that promotes elevated translation elongation, through modulation of eEF2K/eEF2 signalling, to drive progression of a mouse CLL-like disease. The use of selective mTOR inhibitors on proliferating primary CLL and mouse CLL cells has highlighted the potential to inhibit translation elongation processes, and identifies a novel therapeutic target for patients with progressive CLL downstream of mTORC1.

### Supplementary information

Supplemental Data File
