# Supplementary material for: mTORC1-selective activation of translation elongation promotes disease progression in chronic lymphocytic leukemia
Source: Leukemia. 2023 Sep 29;37(12):2414–25. doi: 10.1038/s41375-023-02043-3 (PMC10681897; doi:10.1038/s41375-023-02043-3)

**Supplemental Data File for:**

**mTORC1-selective activation of translation elongation promotes disease progression in chronic lymphocytic leukemia**

Natasha Malik^1^, Jodie Hay^1^, Hassan N.B. Almuhana^1^, Karen M. Dunn^1^, Jamie Lees^1^, Jennifer Cassels^1^, Jiatian Li^1^, Rinako Nakagawa^2^, Owen J. Sansom^1,2^, Alison M. Michie^1^

^1^Institute of Cancer Sciences, College of Medicine, Veterinary and Life Sciences,

University of Glasgow, Glasgow UK; ^2^Cancer Research UK Beatson Institute, Garscube Estate, Glasgow, UK. ^3^Cancer Research UK Beatson Institute; Garscube Estate, Glasgow, UK.

Supplementary Methods:

*Cell Culture conditions:* Primary CLL cells and the MEC1 CLL cell line were cultured at 1x10^6^/mL in RPMI-1640 containing 10% foetal bovine serum (FBS), 50 U/mL penicillin, 50 mg/mL streptomycin, and 2 mM L-glutamine (CLL medium; Invitrogen Ltd., Paisley, UK). OP9 stromal cells were maintained in OP9 medium (αMEM medium supplemented with 20% FBS, 50 U/mL penicillin, 50 mg/mL streptomycin, 2 mM L-glutamine, 10 mM HEPES, 1 mM sodium pyruvate, 10 µg/ml gentamycin, 50 µM βME). The fibroblast cell line constitutively expressing CD40L (NT-L-CD40L) was maintained in CLL medium. All cells were cultured at 37ºC in a humidified incubator with 5% (v/v) CO_2_. Cell lines were authenticated based on functional capacity, morphology and/or phenotype, and were tested for the mycoplasma contamination once every 6-8 months.

*In vitro OP9 co-culture of retrovirally-transduced HPCs:* LSKs were retrovirally-transduced with either empty vector (MIEV) or kinase dead PKCα (PKCα-KR) vector to induce a CLL-like disease.^1^ Cells were co-cultured with the OP9 cell line supplemented with IL-7 and Flt-3 (each 10 ng/mL) until day 7-10, passaging every 2-3 days. After d10, cells were further co-cultured with OP9 and IL-7 only. To induce *Raptor-*excision *in vitro,* retrovirally-transduced HPCs from *Mx1*-*Raptor* mice were co-cultured with OP9 cells *in vitro* until d10. Then 0.5-1x10^6^ cells/well were treated with 200 U/well IFNβ for 24 hr to excise *Raptor*. The cells were harvested 4 days post treatment and used in ongoing experiments, as indicated.

*Migration Assay*: *CD19-Raptor*-PKCα-KR cells (2-5x10^6^ cells/ml) were cultured for 2 hr at 37ºC in resting medium (DMEM containing 0.5% bovine serum albumin (BSA), 10 mM HEPES, 1 mM sodium pyruvate, 100 µg/ml streptomycin, 100 U/ml penicillin, 2 mM L-glutamine). Resting medium was supplemented with 150 ng/ml SDF-1 (PeproTech, UK) to make migration medium. Transwell^®^ permeable support chambers (6.5 mm-diameter upper chamber, pore diameter 5 μm transwell culture insert; Corning Inc, ME, USA) were set up such that the bottom contained 600 µl migration medium and 100 µl cells from the resting step was pipetted onto the top chamber. Each condition was carried out in technical duplicates. For negative and positive controls, resting medium was used instead of the migration medium: rested cells were either pipetted onto the chamber or directly into the bottom respectively. Cells were cultured at 37ºC for 4 hr. 150 µl medium was pipetted from every well in technical triplicate and counted on the flow cytometer on low for 30 sec. Data shown is an average of 3 individual mice.

*Primary CLL proliferation co-cultures*: CLL cells were co-cultured with the NTL-CD40L cell line at a ratio of 1:75, as described previously.^2,3^ The culture was supplemented with 15 ng/mL IL-21 (PeproTech, UK) to induce proliferation for 5-8 days. These co-cultures were treated with DMSO as vehicle/no drug control (NDC), 10 nM rapamycin (RAP), 100 nM AZD8055 (AZD or A8055), 1 µM ibrutinib (IB), 500 nM AZD2014 (A2014) or RAP/IB or AZD/IB combinations, as indicated.

*Flow Cytometry*: Single cell suspensions from *in vivo* or *in vitro* experiments were prepared for phenotypic analysis by flow cytometry as described previously.^4,5^ All antibodies were purchased from BD Biosciences (Oxford, UK), except CD21 (Clone: 7E9, BioLegend), CD1d (Clone: 1B1, BioLegend), IgD (Clone: 11-26c.2a, BioLegend), CD23 (Clone: B3B4, BioLegend). For cell cycle analysis, treated 1x10^6^ PKCα-KR and 2x10^6^ primary CLL patient cells were fixed and permeabilized by adding 1 ml cold 80% ethanol dropwise to the pellet and stored at -20˚C until analysis. After washing the samples, 350-500 µl propidium iodide (PI)/RNAse Staining Buffer (BD Biosciences) was added to each sample and incubated at RT in the dark for 15 min before analysis. For cell proliferation, cells were stained with CellTrace™ Violet stock solution (Invitrogen, Paisley, UK) as per the manufacturer’s protocol. PKCα-KR cells were acquired for 3 consecutive days (every 24 hr) and primary CLL patient samples were recorded on day 5-8 of culture to assess proliferation. For apoptosis analysis, treated 5x10^5^ PKCα-KR or primary CLL patient cells were washed with 1x HBSS (ThermoFisher Scientific) at 300*g* for 5 min at RT. Cells were incubated in 100 µl HBSS containing 2.5 µl Annexin V and 2.5 µl 7AAD (BD Biosciences) for 10 min at RT in the dark and analysed. Data were acquired on a FACSCantoII with BD FACSDiva software and analysed using FlowJo software (Tree Star Inc., OR).

*qPCR*: RNA was extracted from fresh cells following the RNeasy Qiagen Kit protocol. cDNA was made using standard protocols (Invitrogen). qPCR was carried out on a 384 well plate in triplicate by the 7900HT Fast Real-Time PCR system (Applied Biosystems, Warrington, UK). The primers used are listed in Table S2. *Raptor* excision PCR: Successful recombination by Cre recombinase (using *Rptor* for and *Rptor* del primers) resulted in a 204bp product (del).^6^ In brief, 240 ng/25 μl of DNA was amplified in a PCR reaction with a hot start of 95°C for 2 min followed by 40 cycles of: 30 sec denaturation at 95°C, 30 sec annealing at 59°C, 1 min extension at 72°C; and a final extension at 72°C for 5 min, before separating with gel electrophoresis.

*Western blotting*: Protein lysates of cell pellets were prepared in lysis buffer (20 mM Tris pH 7.4, 2mM EDTA, 1% Triton, 1mM DTT) containing protease inhibitor cocktail and phosphatase inhibitor cocktail (Roche) and incubated on ice for 30 min. Bradford assay was performed to quantify protein samples using a standard protocol (Bio-Rad). 4-12% pre-cast gels (NuPAGE Novex BisTris) were used for gel electrophoresis. Western blotting was performed by following the standard protocol provided (Invitrogen). The PVDF membranes were blocked in 5% milk and primary antibodies used were prepared in 1% BSA. List of antibodies are listed in Table S3. Signals were detected using Imobilon Forte HRP substrate (Millipore).

*
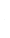
Protein Synthesis Assay*: The O-Propargyl-puromycin (OPP) incorporation was performed as part of the Global Protein Synthesis Assay (Abcam), as per manufacturer’s instructions. In brief, following drug treatments, 1x10^5^ cells/100 μL were transferred to a 96 well plate. A control well was incubated with 1X cycloheximide for 1 hr. All samples were the incubated with 1X Protein label for 1 hr before fixing and permeabilising the cells and then staining with the reaction protocol as outlined in the protocol. Samples were analysed by flow cytometry for the amount of fluorescence generated by *de novo* synthesized protein during the Click reaction

*MEC1 Transient Transfection:* Raptor knockdown in MEC1 cells was performed by delivery of an shRNA construct targeting Raptor (MISSION® shRNA, Sigma Aldrich) with Lipofectamine 2000 reagent (Thermo Fisher Scientific). In brief, DNA-lipid complex was prepared with plasmid DNA targeting Raptor or scrambled control in a 1:5 ratio (1 μg DNA: 5 μl transfection reagent) to 1x10^6^ MEC1 cells in a 48 well plate. Cells were incubated for 3 days prior to experimental set up.

**References**

1. Nakagawa R, Soh JW, Michie AM. Subversion of Protein Kinase Cα Signaling in Hematopoietic Progenitor Cells Results in the Generation of a B-Cell Chronic Lymphocytic Leukemia–Like Population In vivo. *Cancer Res*. 2006;66(1):527–34.

2. McCaig AM, Cosimo E, Leach M, Michie AM. Dasatinib inhibits CXCR4 signaling in chronic lymphocytic leukemia cells and impairs migration towards CXCL12. PLos One. 2012;7:e48929.

3. Pascutti MF, Jak M, Tromp JM, Derks IAM, Remmerswaal EBM, Thijssen R, et al. IL-21 and CD40L signals from autologous T cells can induce antigen-independent proliferation of CLL cells. Blood. 2013;122:3010-9.

4. Malik N, Dunn K, Cassels J, Hay J, Estell C, Sansom OJ, et al. mTORC1 activity is essential for erythropoiesis and B cell lineage commitment. Sci Rep. 2019;9:16917.

5. Cosimo E, Tarafdar A, Moles MW, Holroyd AK, Malik N, Catherwood MA, et al. AKT/mTORC2 Inhibition Activates FOXO1 Function in CLL Cells Reducing B-Cell Receptor-Mediated Survival. Clin Cancer Res. 2019 Mar;25(5):1574–87.

6. Bentzinger CF, Romanino K, Cloëtta D, Lin S, Mascarenhas JB, Oliveri F, et al. Skeletal Muscle-Specific Ablation of raptor, but Not of rictor, Causes Metabolic Changes and Results in Muscle Dystrophy. *Cell Metab.* 2008;8(5):411–24.

| **CLL ID** | **Treatment^a^** | **Sex** | **Binet Stage** | **ZAP-70 status^b^** | **Cytogenetics** |
| --- | --- | --- | --- | --- | --- |
| 9 | No | F | A | neg | No 11q/17p |
| 113 | Yes | F | C | high | del17p |
| 116 | No | M | A | high | No 11q/17p |
| 140 | No | F | A | low | T12 |
| 147 | No | M | C | high | No 11q/17p |
| 151 | No | M | B | ND | del11q |
| 165 | No | M | C | ND | del11q |
| 168 | Yes | M | B | ND | del13q, T12 |
| 169 | No | F | C | ND | del11q |
| 171 | No | M | C | ND | No 11q/17p |
| 173 | Yes | M | B | ND | del11q |
| 175 | Yes | M | C | ND | No 11q/17p |
| 176 | No | M | A | ND | No 11q/17p |
| 177 | No | M | B | ND | No 11q/17p |
| 179 | Yes | M | B | ND | No 11q/17p |
| 180 | Yes | F | B | ND | No 11q/17p |
| 185 | Yes | M | C | ND | ND |
| 186 | No | M | C | ND | ND |
| 187 | No | M | B | ND | ND |
| 189 | Yes | M | B | ND | No 11q/17p |
| 190 | No | M | C | ND | No 11q/17p |
| 191 | No | M | A | ND | del11q, del17p |
| 193 | No | F | A | ND | No 11q/17p |
| 194 | No | M | C | ND | ND |

Table S1. CLL patient clinical characteristics.

^a^ If previously undergone treatment, it was not within three months of sample collection.
^b^ ZAP-70 analysis was conducted by immunohistochemistry in the regional haematology laboratory. ND – not determined.

| Gene | Forward | Reverse | Species | Sequence Information |
| --- | --- | --- | --- | --- |
| *Ebf1* | tacagaaggtcattcctcgg | atcccatacagggcttcaac | Mouse | NM_001290709.1 |
| *Pax5* | acagga catggaggag tgaa | tgacaccttg atgggcaagt | Mouse | NM_008782.2 |
| *Rptor* | atggtagcaggcacactcttcatg | gctaaacattcagtccctaatc | Mouse | Ref. 5 |
| *Rptor-del* | ctcagagaactgcagtgctgaagg |  | Mouse | Ref. 5 |
| *Gusb* | taagacgctgatcacccaca | cagataacatccacgtacgg | Mouse | NM_010368.1 |
| *Tbp* | gtacccttcaccaatgactc | cagccaagattcacggtaga | Mouse | NM_013684.3 |

Table S2: List of primers used for PCR reactions.

The full sequence for each gene was obtained from the NCBI gene database (https://ncbi.nlm.nih.gov/gene). Each primer was designed to have close to 10 C=G and 10 A=T bonds. The length between the forward and reverse primer is between 150-300 base pairs. *Gusb* and *Tbp* were used as reference genes for murine cells. The specificity of each primer sequence was checked by using BLASTn tool.

| Name | Named Species Reactivity | Clone | Dilution | 2^nd^ary Ab |
| --- | --- | --- | --- | --- |
| RAPTOR | Human, Mouse, Rat | 24C12 | 1:1000 | Rabbit |
| pAKT^S473^ | Human, Mouse, Rat | D9E | 1:1000 | Rabbit |
| AKT (pan) | Human, Mouse, Rat | C67E7 | 1:1000 | Rabbit |
| pS6^S235/S236^ | Human, Mouse, Rat | D57.2.2E | 1:1000 | Rabbit |
| S6 | Human, Mouse, Rat | 54D2 | 1:1000 | Mouse |
| p4EBP1^T37/T46^ | Human, Mouse, Rat | 236B4 | 1:1000 | Rabbit |
| peEF2^T56^ | Human, Mouse, Rat | #2331 | 1:1000 | Rabbit |
| eEF2 | Human, Mouse, Rat | #2332 | 1:1000 | Rabbit |
| peEF2k^S366^ | Human, Rat, Monkey | #3691 | 1:500 | Rabbit |
| eEF2k | Human, Rat, Monkey | #3692 | 1:500 | Rabbit |
| Cyclin A | Human, Mouse | C-19 | 1:1000 | Rabbit |
| Mcl1 | Human, Mouse | D34A5 | 1:1000 | Rabbit |
| 4EBP1 | Human, Mouse, Rat | 53H11 | 1:1000 | Rabbit |
| GAPDH | Human, Mouse, Rat | D16H11 | 1:1000 | Rabbit |
| β-ACTIN | Human, Mouse, Rat | C4 | 1:1000 | Mouse |
| α-mouse IgG, HRP Ab |  |  | 1:10000 |  |
| α-rabbit IgG, HRP Ab |  |  | 1:10000 |  |

Table S3: List of antibodies used for western blotting.

List of antibodies (Ab) and their dilutions in 5% BSA in TBS-T. All antibodies were purchased from Cell Signalling Technologies (Herts, UK), except Cyclin A and β-Actin (Santa Cruz, USA).


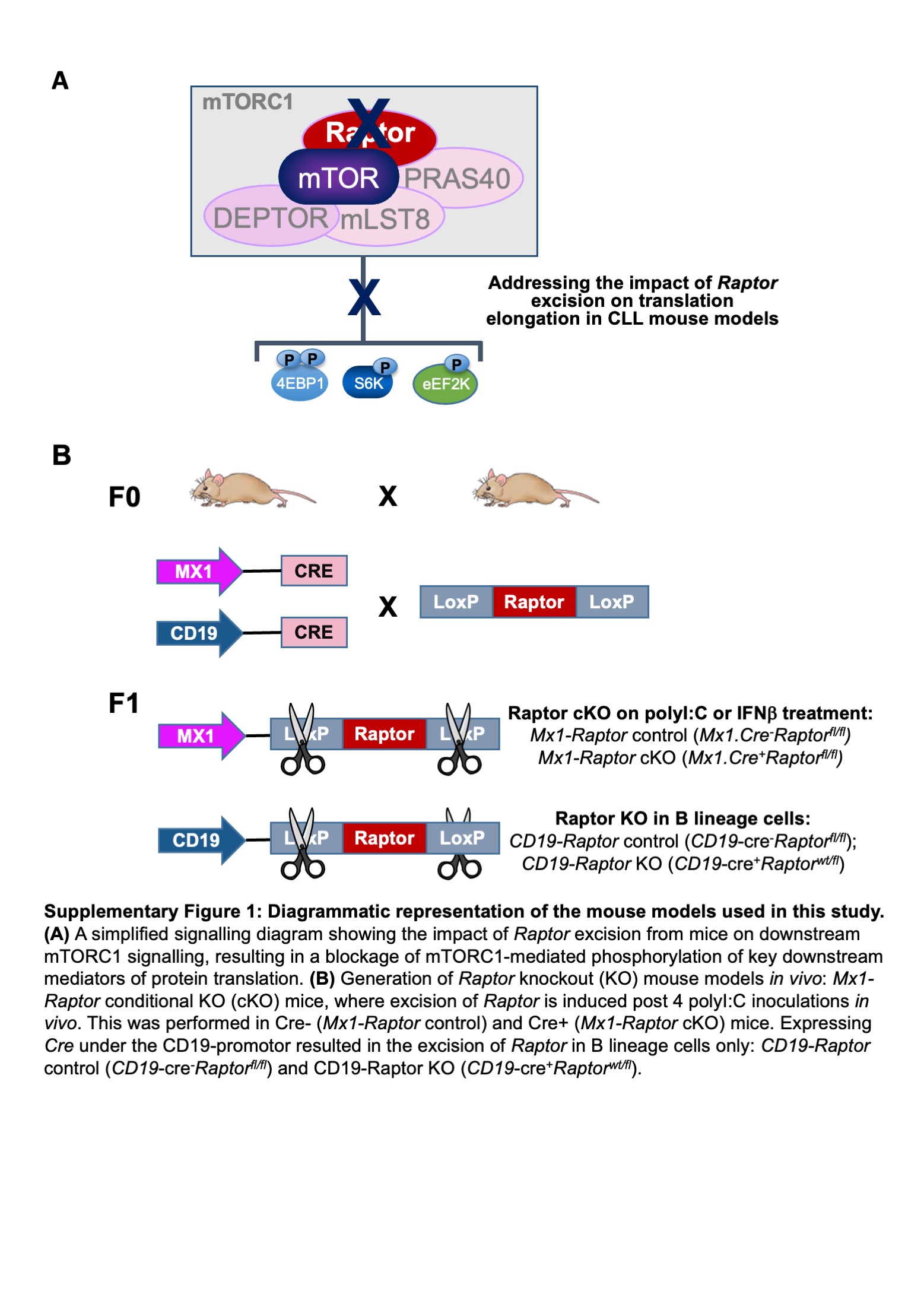


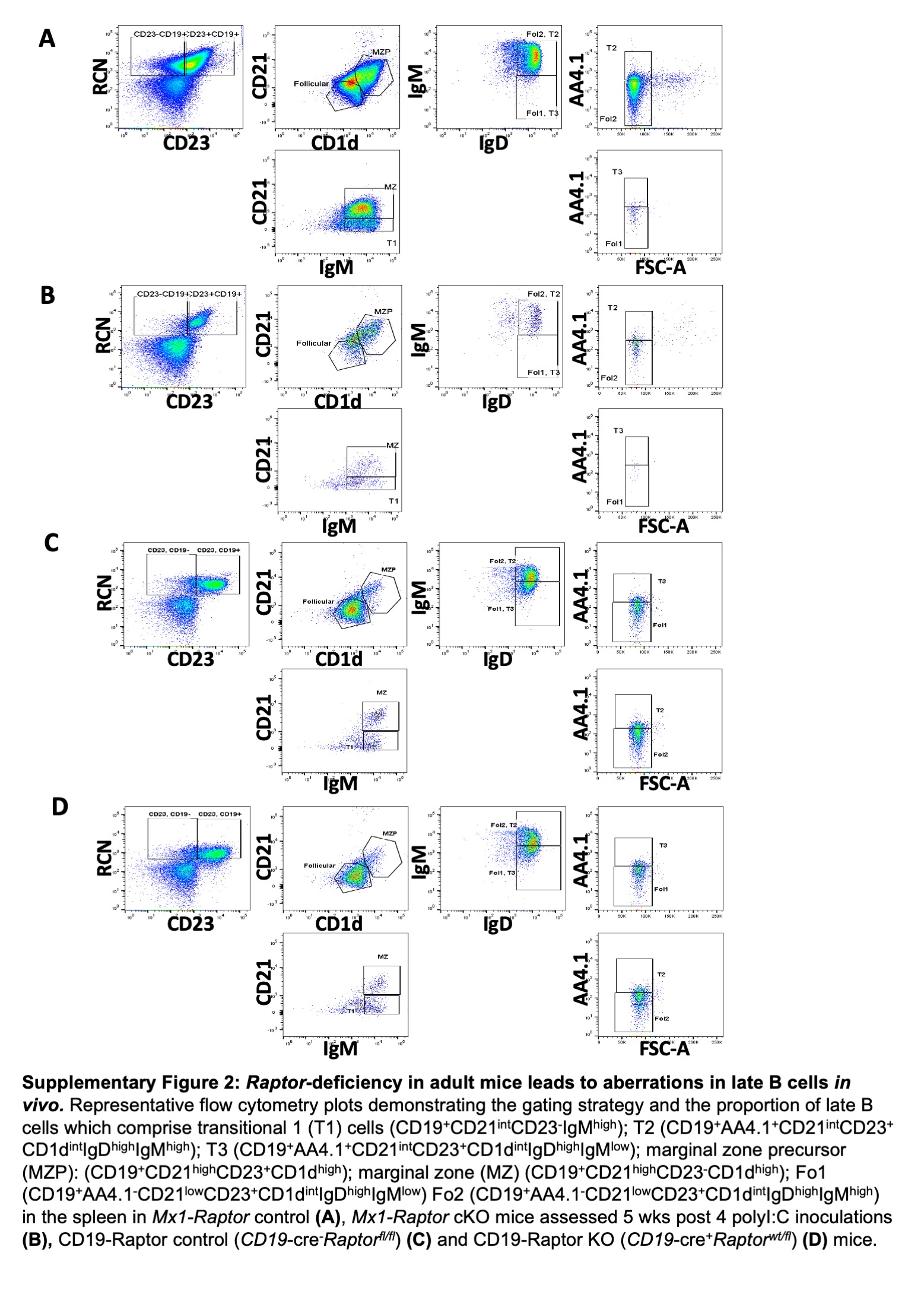


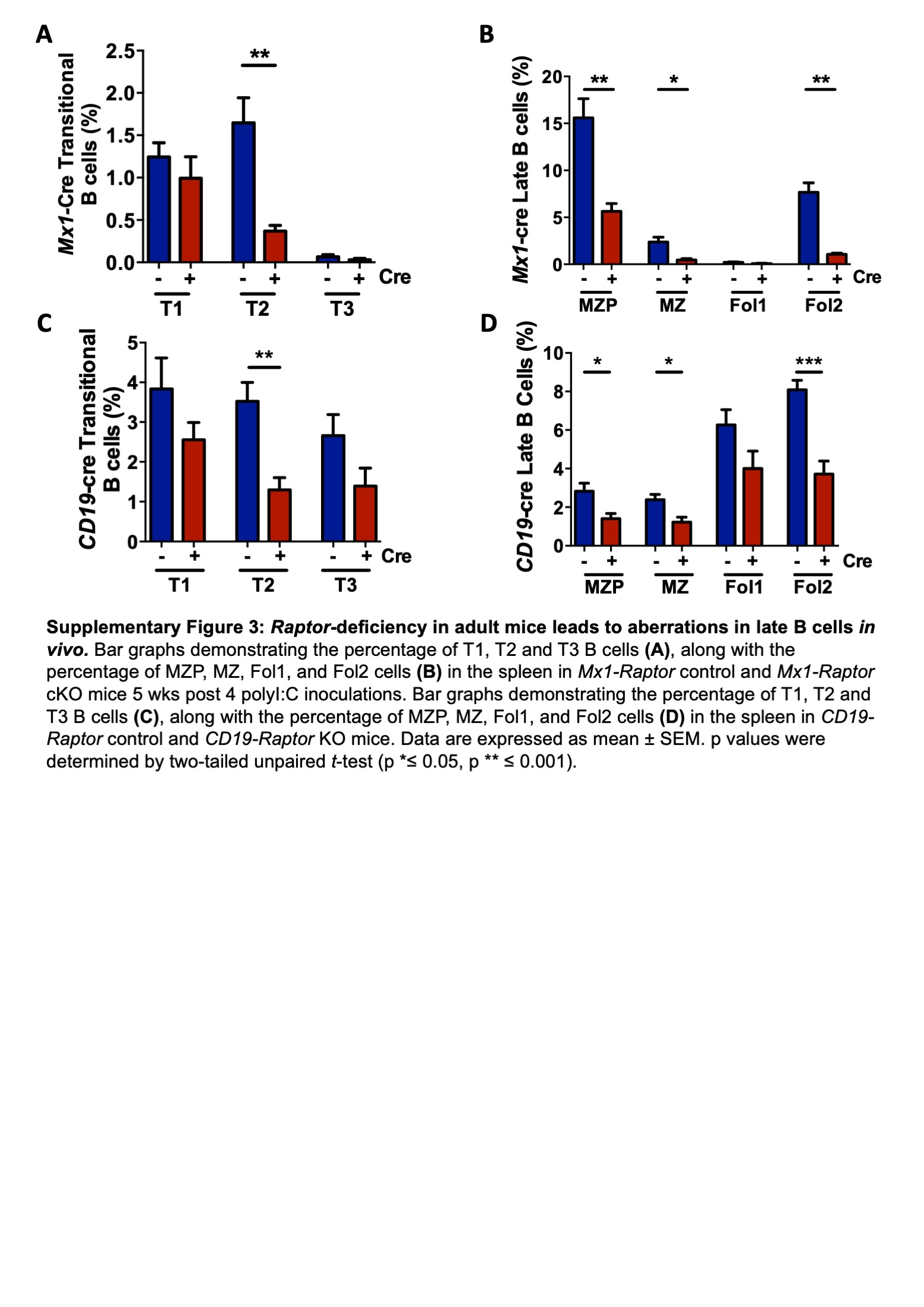


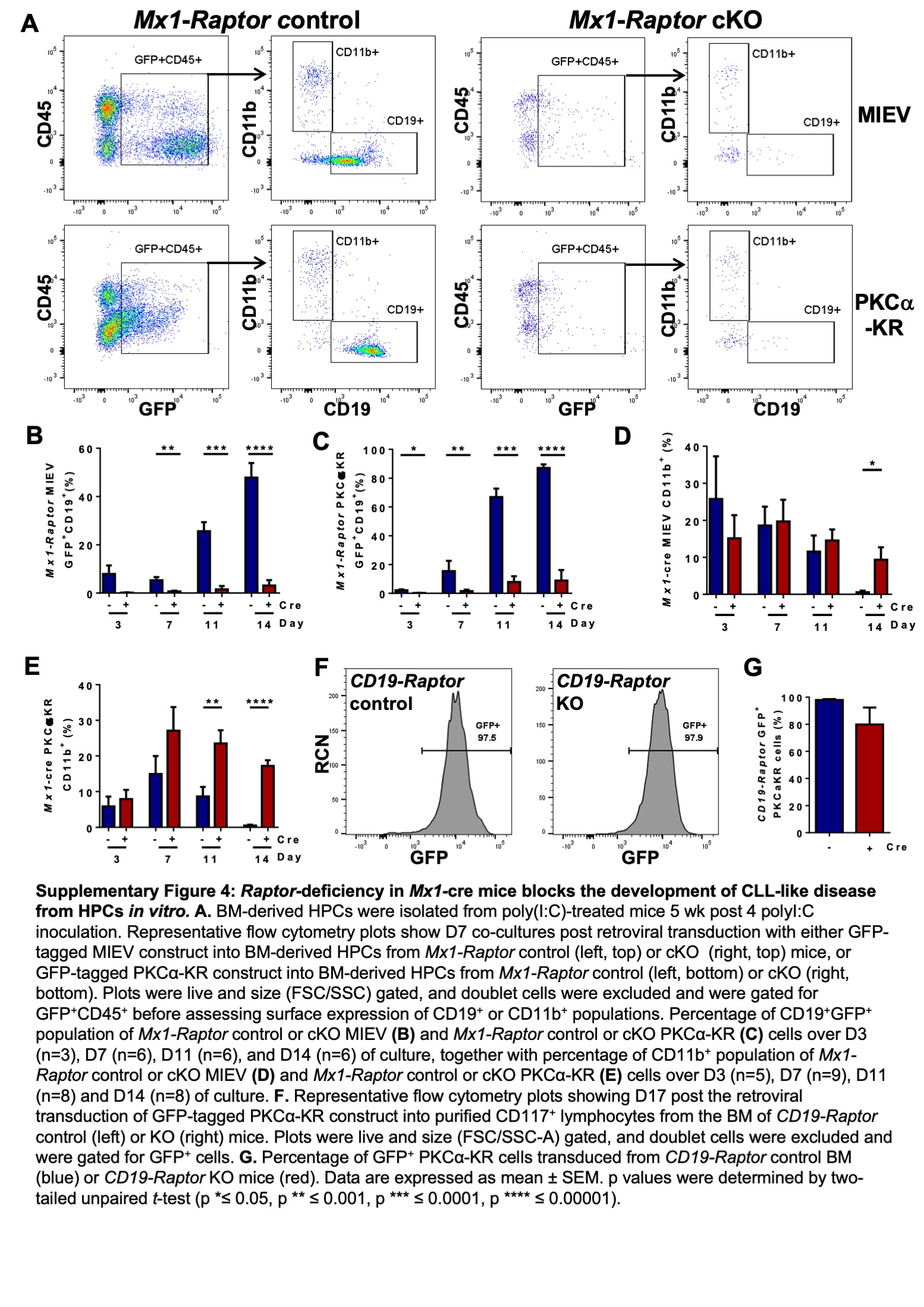


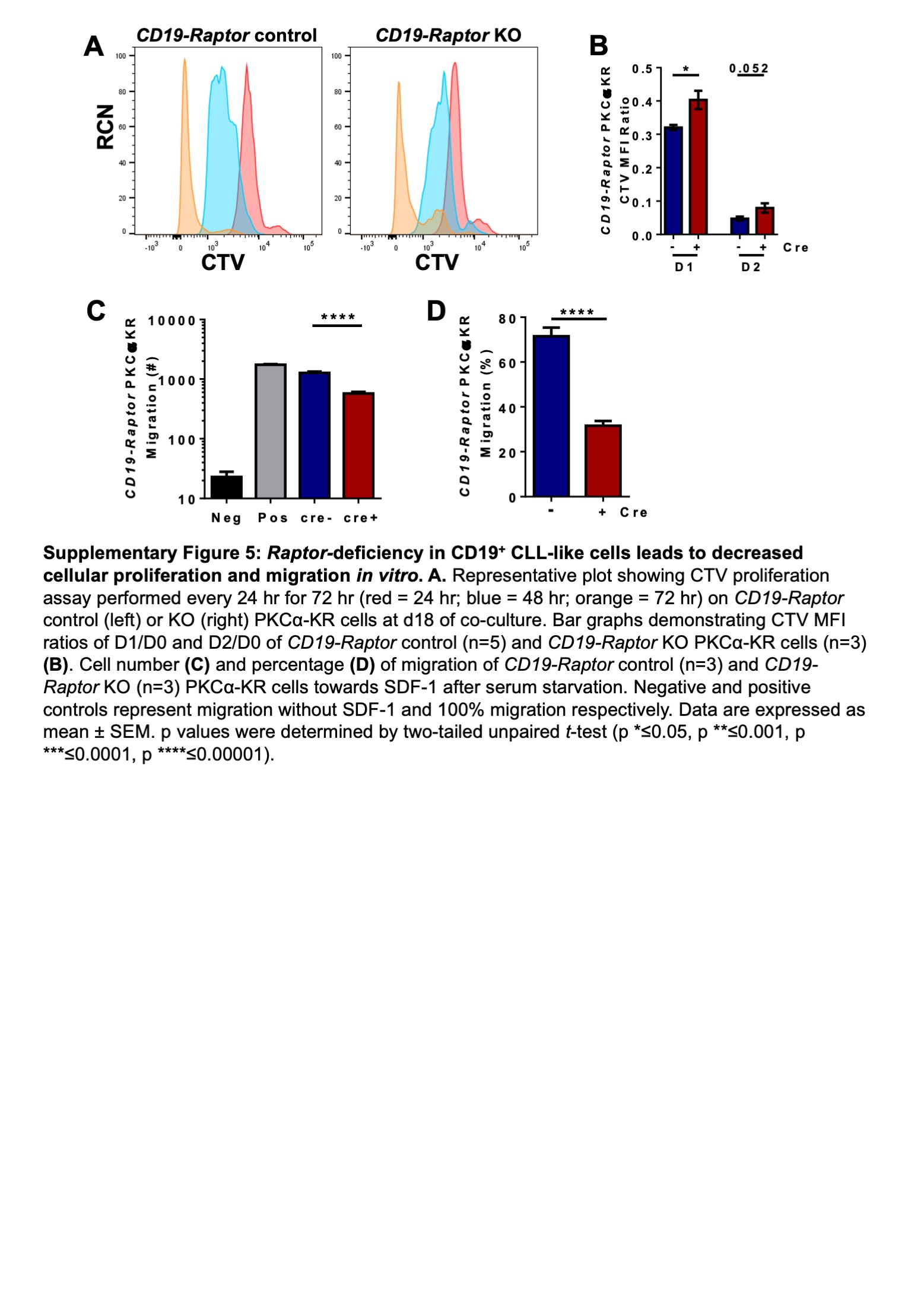


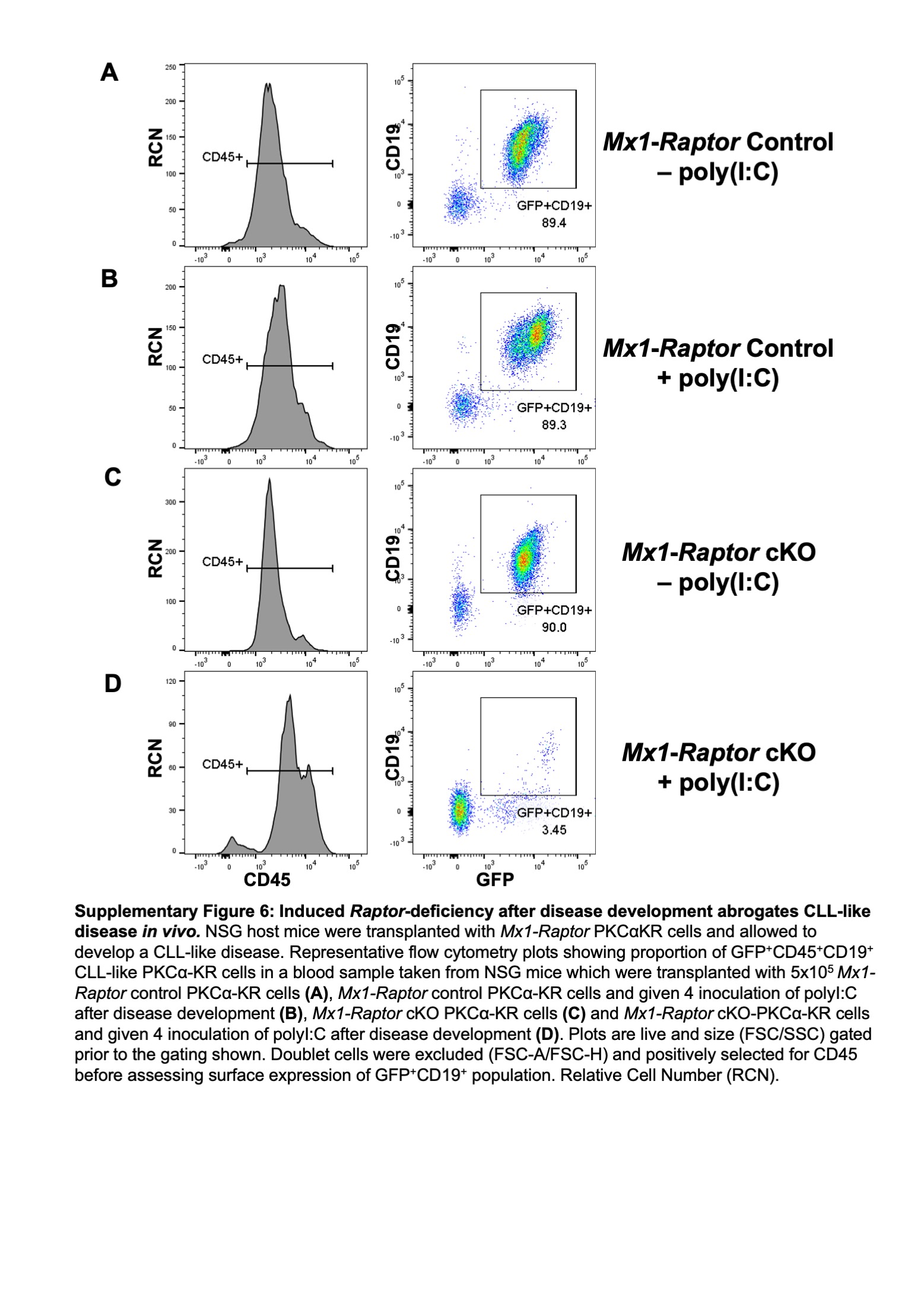


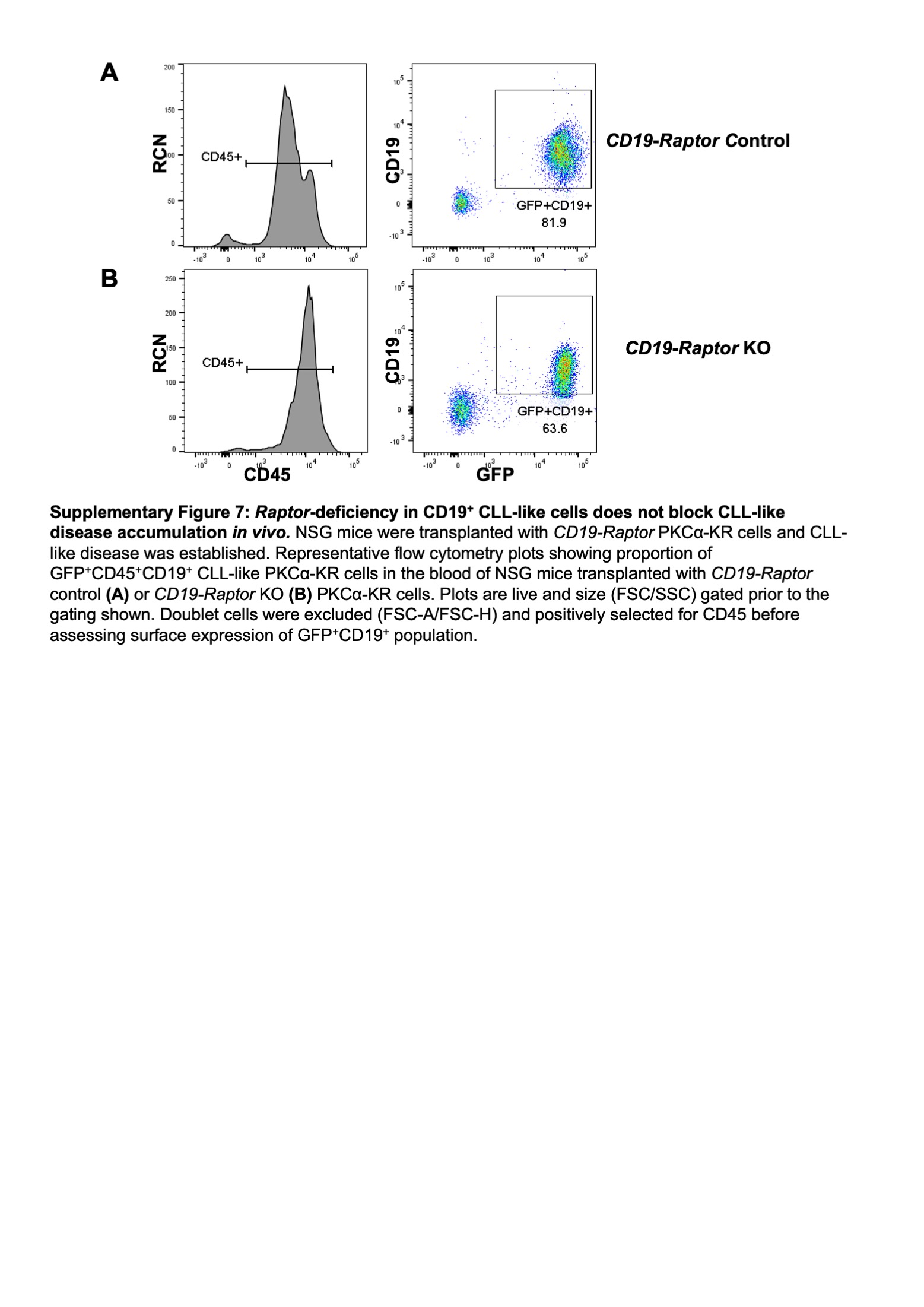


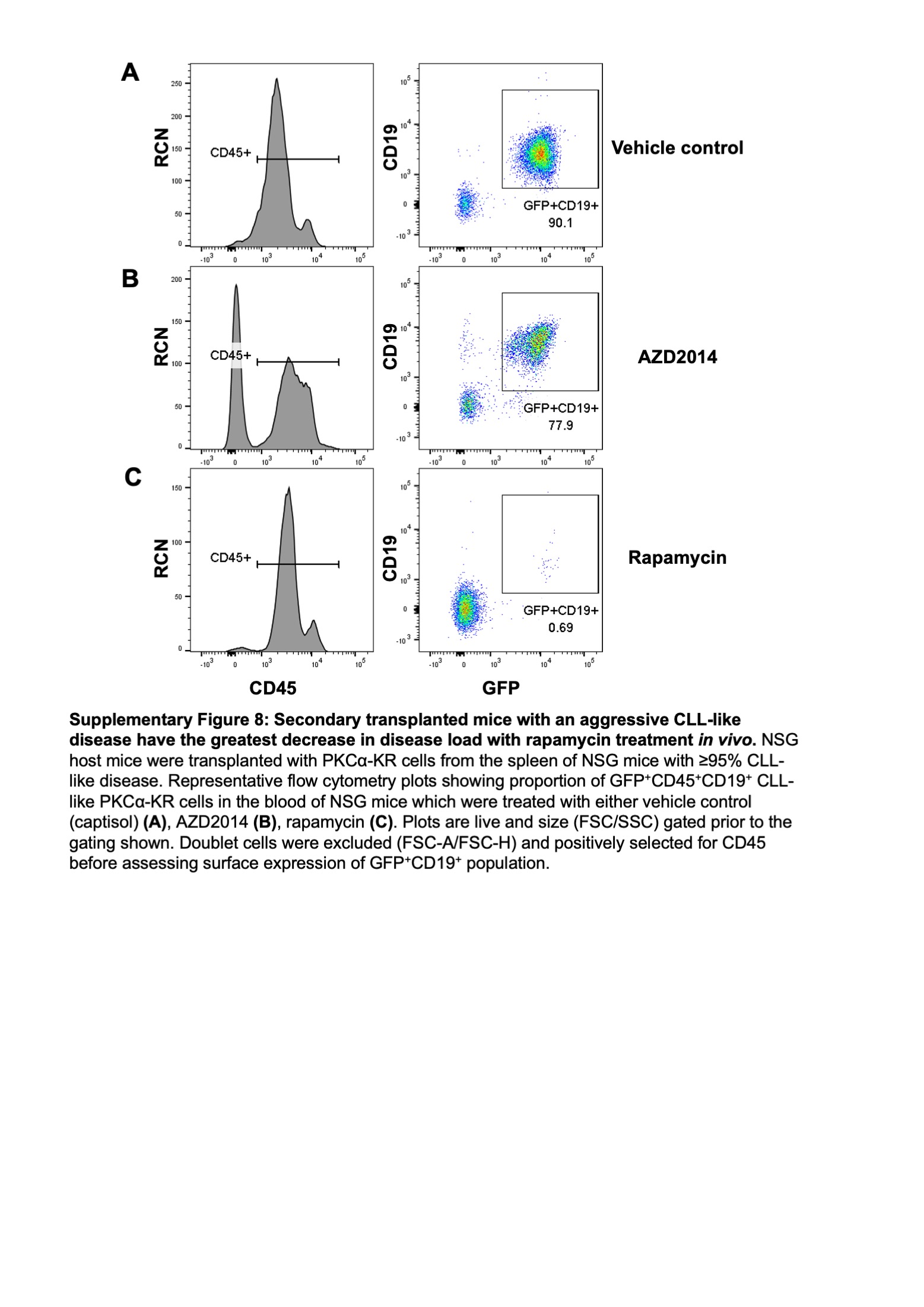


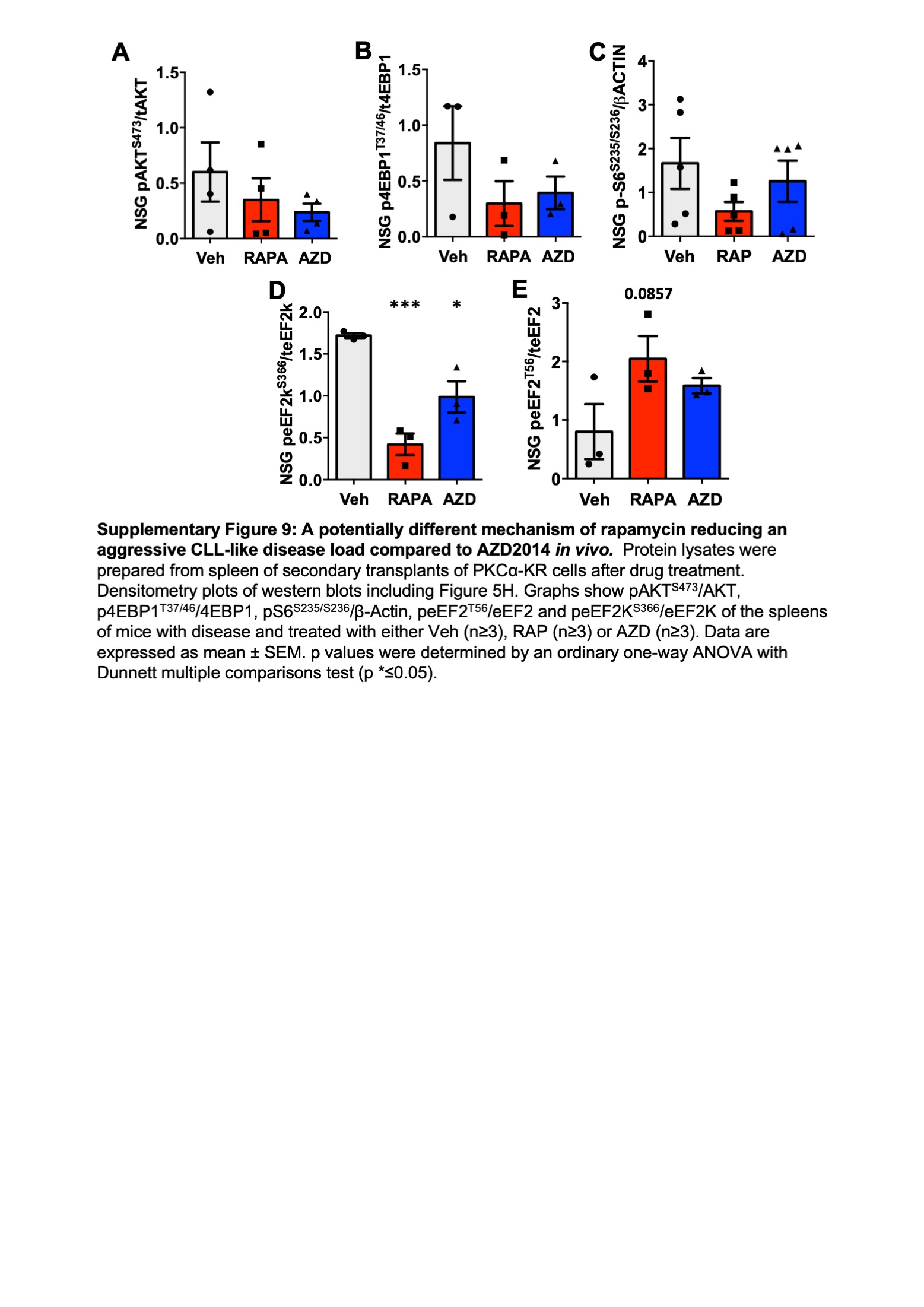


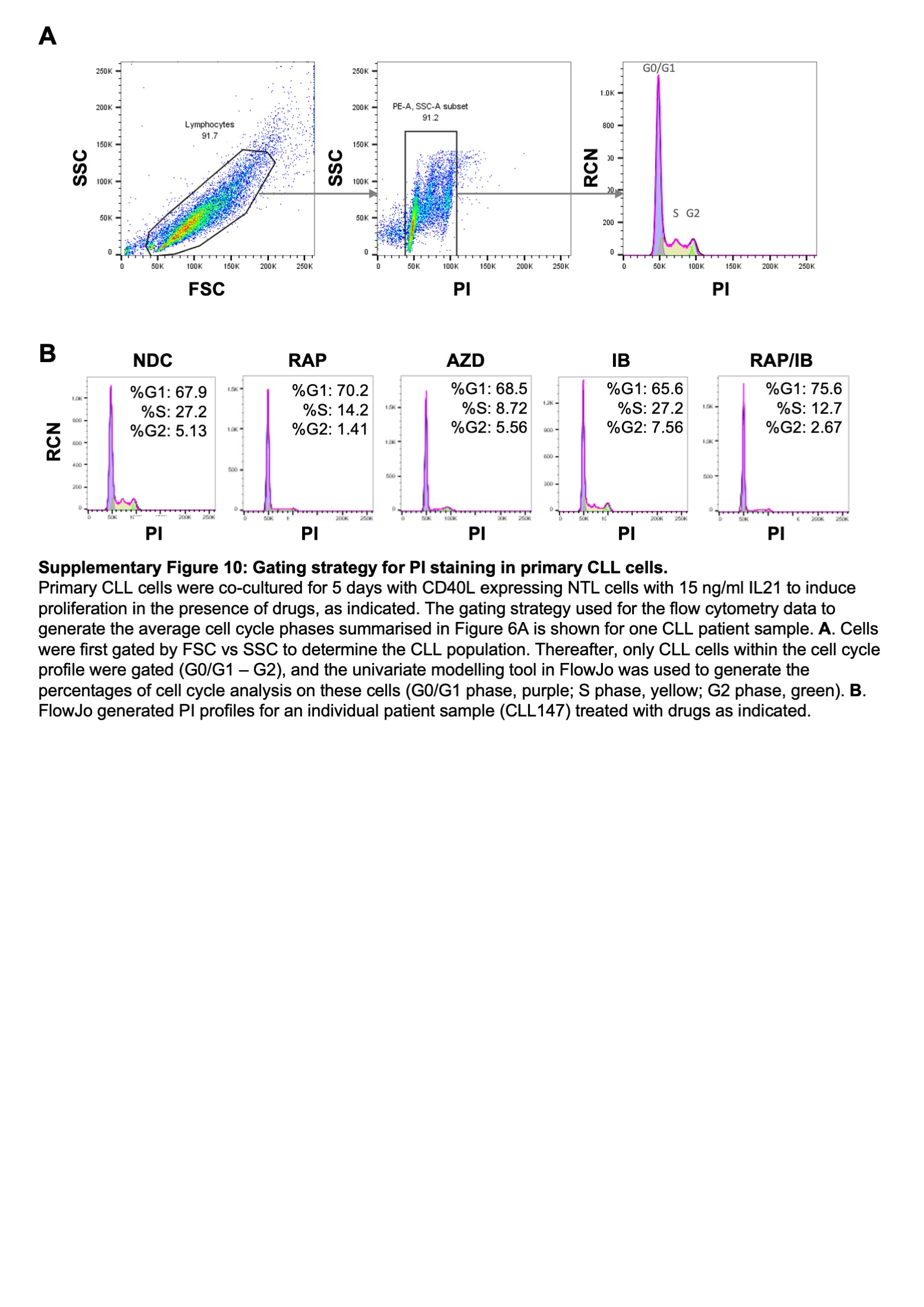


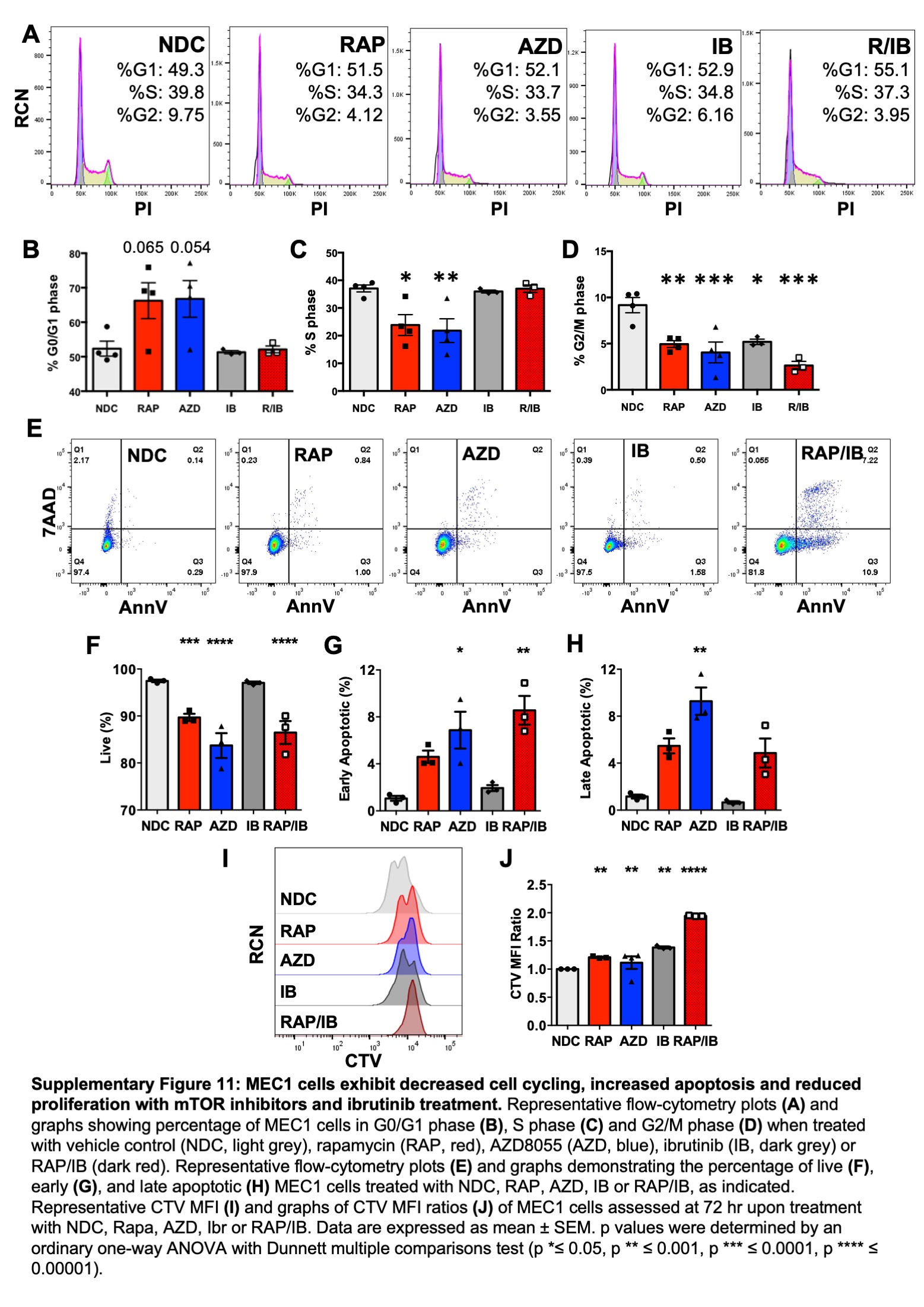


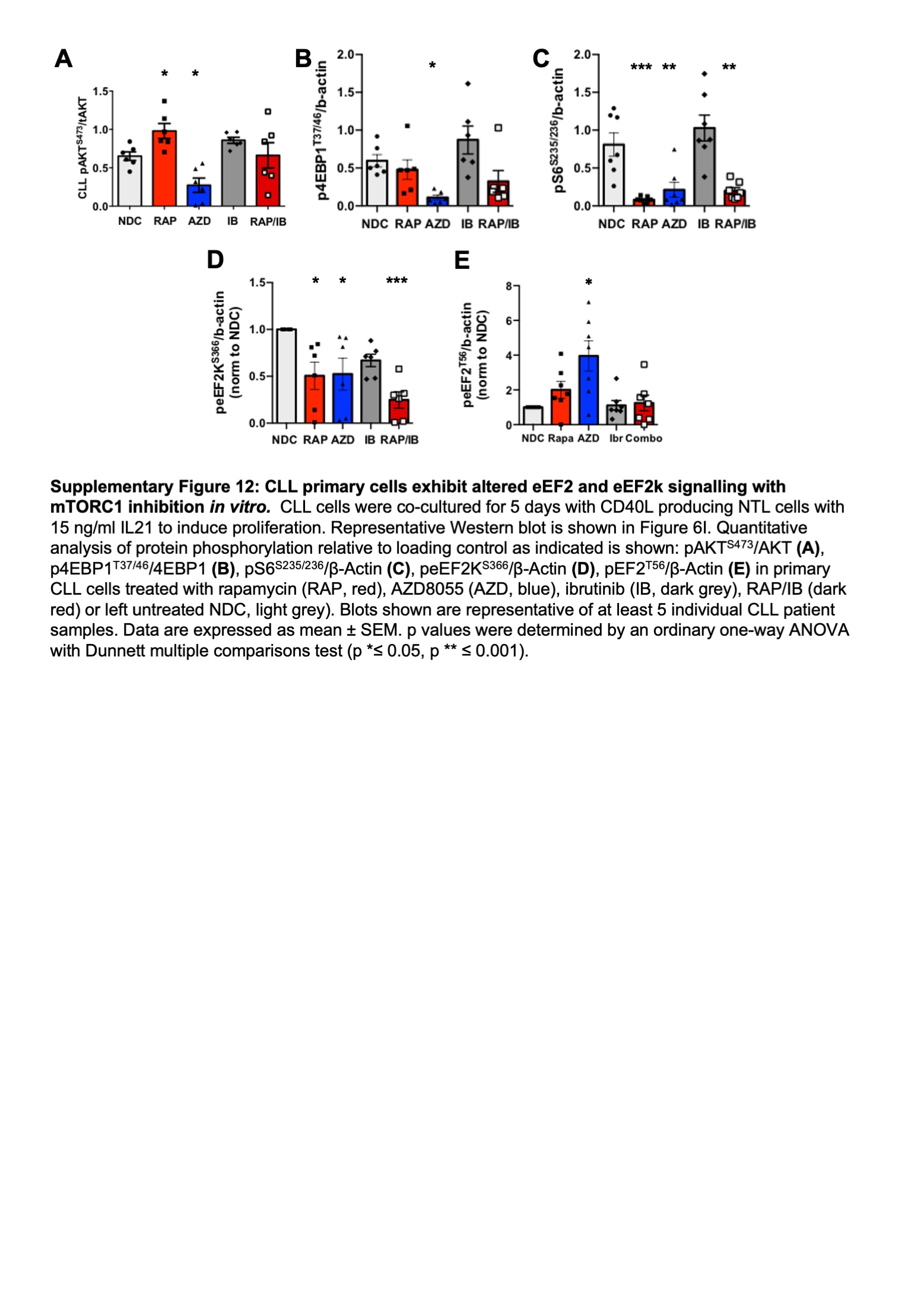


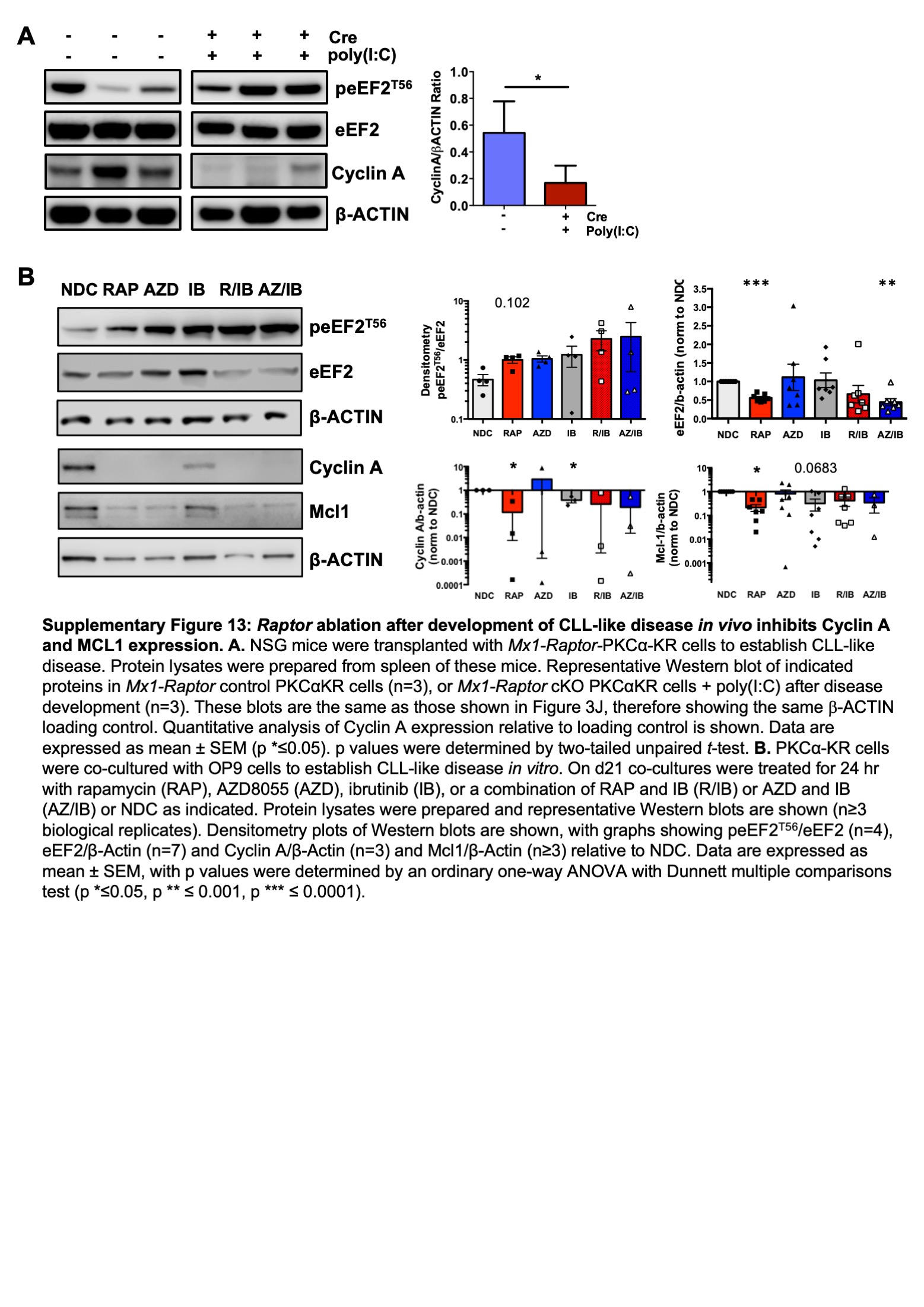


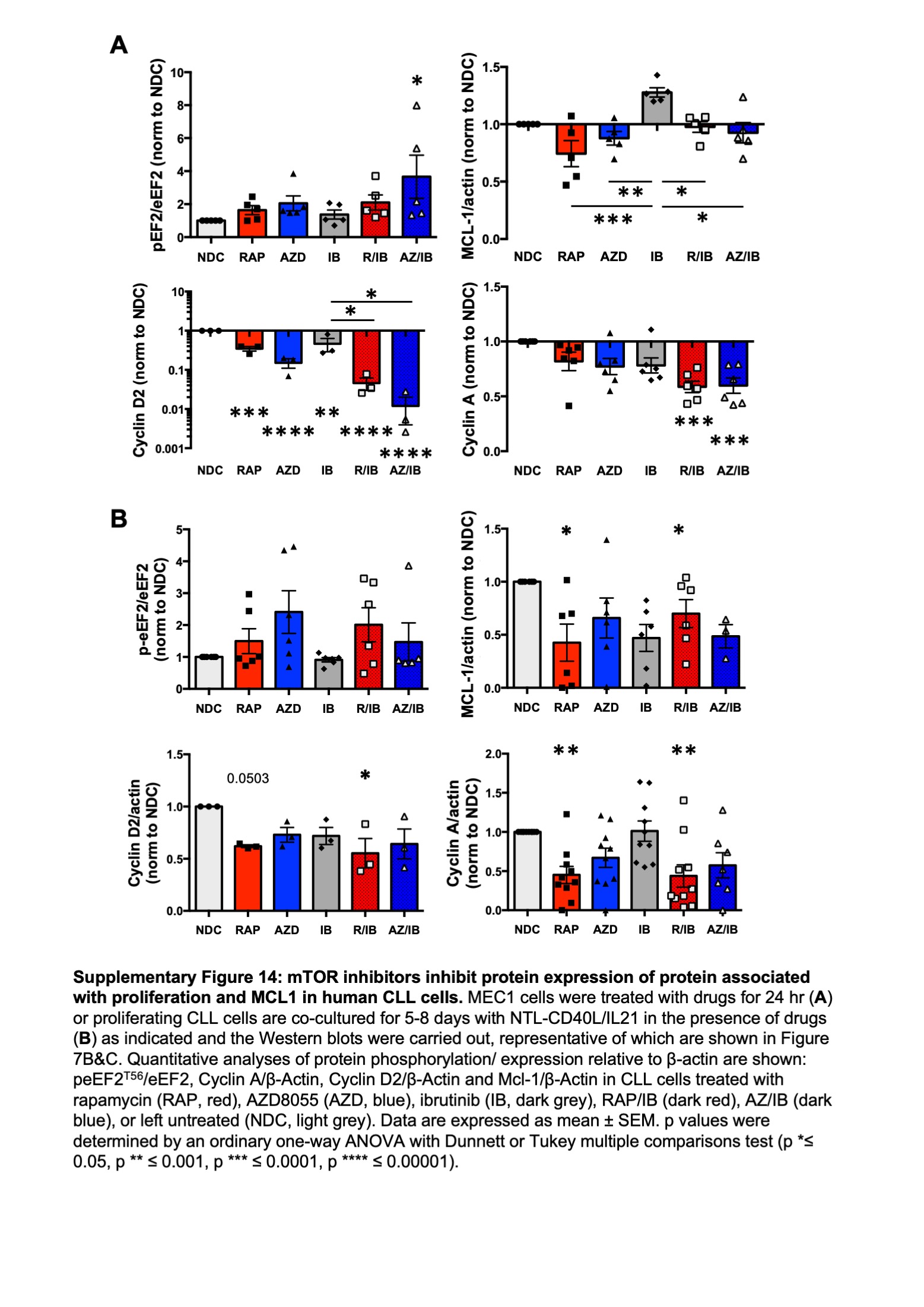


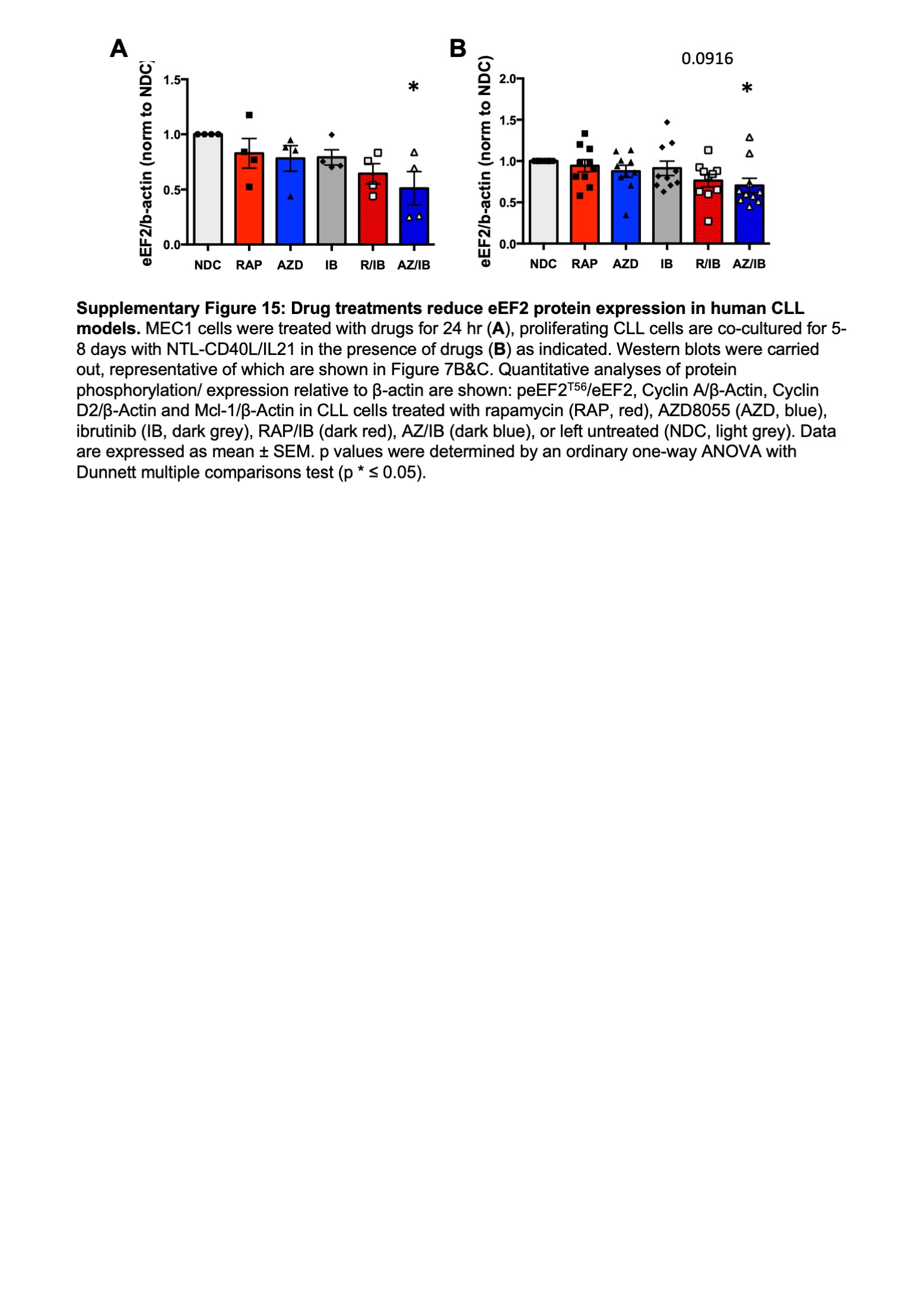

Supplement: Supplementary file 1 — Supplemental Data File [file 41375_2023_2043_MOESM1_ESM.docx]
